# Structure and Ionic Conductivity of Li-Disordered Bismuth *o*-Thiophosphate Li_60–3*x*_Bi_16+*x*_(PS_4_)_36_

**DOI:** 10.1021/acs.inorgchem.3c01028

**Published:** 2023-06-29

**Authors:** Maximilian A. Plass, Maxwell W. Terban, Tanja Scholz, Igor Moudrakovski, Viola Duppel, Robert E. Dinnebier, Bettina V. Lotsch

**Affiliations:** † Max Planck Institute for Solid State Research, Heisenbergstraße 1, 70569 Stuttgart, Germany; ‡ University of Munich (LMU), Butenandtstraße 5-13, 81377 München, Germany

## Abstract

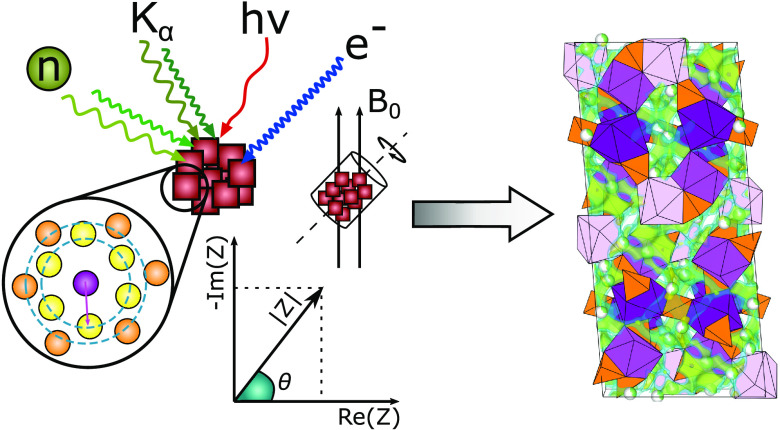

The structure of the first lithium-containing bismuth ortho (*o*)-thiophosphate was determined using a combination of powder X-ray, neutron, and electron diffraction. Li_60–3*x*_Bi_16+*x*_(PS_4_)_36_ with *x* in the range of 4.1–6.5 possesses a complex monoclinic structure [space group *C*2/*c* (No. 15)] and a large unit cell with the lattice parameters *a* = 15.4866 Å, *b* = 10.3232 Å, *c* = 33.8046 Å, and β = 85.395° for Li_44.4_Bi_21.2_(PS_4_)_36_, in agreement with the structure as observed by X-ray and neutron pair distribution function analysis. The disordered distribution of lithium ions within the interstices of the dense host structure and the Li ion dynamics and diffusion pathways have been investigated by solid-state nuclear magnetic resonance (NMR) spectroscopy, pulsed field gradient NMR diffusion measurements, and bond valence sum calculations. The total lithium ion conductivities range from 2.6 × 10^–7^ to 2.8 × 10^–6^ S cm^–1^ at 20 °C with activation energies between 0.29 and 0.32 eV, depending on the bismuth content. Despite the highly disordered nature of lithium ions in Li_60–3*x*_Bi_16+*x*_(PS_4_)_36_, the underlying dense host framework appears to limit the dimensionality of the lithium diffusion pathways and emphasizes once more the necessity of a close inspection of the structure–property relationships in solid electrolytes.

## Abstract 2

Different analytical techniques were used to characterize the structure and to investigate the ionic transport properties. The combination of these techniques allowed us to elucidate the complex structure of the first lithium-containing bismuth *o*-thiophosphate and provide insights into the electrochemical and ionic diffusion properties.

## Introduction

The discovery of new materials is crucial for improving the performance of rechargeable energy devices such as lithium ion batteries (LIBs) used in hybrid or electric vehicle applications and to enhance their competitiveness with combustion engine vehicles. While liquid organic electrolytes are widely used in most commercial LIBs, all-solid-state batteries (ASSBs) composed of solid electrolytes are increasingly studied due to their advantageous properties such as nonflammability, high transference numbers, increased volumetric energy densities, and simplified cell architectures.^1,2^

Sulfides and thiophosphates are among the most promising solid inorganic electrolyte (SIE) candidates due to their comparatively high ionic conductivities^3−6^ of ≤10^–2^ S cm^–1^ and their mechanical softness, which make grain boundary-related losses less dominant. Another critical aspect of the development of new energy materials is the abundance and price of the raw materials^7−9^ used for their fabrication. While the most critical component in LIBs is usually lithium,^10^ the use of additional expensive elements or components for the large-scale synthesis of high-performance solid electrolytes (SEs) should be avoided to make ASSBs competitive for large-scale applications. In this context, bismuth can be a cheaper alternative compared to germanium or tin.^7−9,11^

Among all of the reported thiophosphates, only a few quaternary compounds containing bismuth have been reported so far. In terms of *o*-thiophosphates, only A_3_Bi(PS_4_)_2_ with A = K, Rb, Cs,^12^ Ag,^13^ or Tl,^14^ K_1.5_Bi_2.5_(PS_4_)_3_,^15^ Cs_3_Bi_2_(PS_4_)_3_,^12^ K_9_Bi(PS_4_)_4_,^15^ and A_3_Bi_3_(PS_4_)_4_ with A = K^15^ or Tl^14^ have been reported. The number of known bismuth hexathiohypodiphosphates is even smaller. Here, only Na_0.16_Bi_1.28_P_2_S_6_^12^ and ABiP_2_S_6_ with A = K,^16^ Ag,^17^ and Tl^14^ have been reported to date. Among bismuth pyrothiophosphates, only three compounds with a composition of ABiP_2_S_7_ with A = K, Rb,^12^ and Tl^14^ and Tl_2_BiP_2_S_7_^18^ are known.

Here, we report on the synthesis and characterization of the first lithium-containing quaternary bismuth *o*-thiophosphate Li_60–3*x*_Bi_16+*x*_(PS_4_)_36_ showing a phase width of 4.1–6.5 for *x*. The structure of this new disordered lithium thiophosphate was determined with a combination of different powder diffraction techniques, and the diffusivity of the disordered lithium ions within the structure has been investigated using electrochemical impedance spectroscopy (EIS), pulsed field gradient nuclear magnetic resonance (PFG-NMR) spectroscopy, and bond valence sum (BVS) analysis.^a^

## Experimental Section

### Synthesis

Li_60–3*x*_Bi_16+*x*_(PS_4_)_36_ was synthesized by mixing Li_2_S (Alfa Aesar, 99.9%), Bi (ChemPur, 99.99%, powder), and P_4_S_10_ (ChemPur, 98%) in stoichiometric ratios and adding a small excess of S_8_ (Merck, sublimed) in an agate mortar in an argon-filled glovebox. The reaction mixture was added to a glassy carbon crucible, transferred into a quartz ampule, and sealed under dynamic vacuum. The quartz ampules were heated under dynamic vacuum using a blow torch and flushed with argon several times to remove traces of O_2_ and H_2_O prior to use. The ampules were then heated to 700 °C for 24 h at a rate of 50 °C  h^–1^ and cooled to room temperature at the same rate.

### Transmission Electron Microscopy

Bulk samples were ground, distributed onto a holey carbon/copper grid, and transferred into the microscope under a protective atmosphere. Transmission electron microscopy (TEM) was performed on a Philips CM 30 ST microscope (300 kV, LaB_6_ cathode) equipped with a spinning star device enabling the use of precession electron diffraction (PED).^20^ Simulations of the diffraction patterns were obtained with the JEMS software package.^21^

### Raman Spectroscopy

Raman spectra of solid-state synthesized powders sealed in quartz capillaries were recorded using a Jobin Yvon Typ V 010 LabRAM single-grate spectrometer equipped with a double super razor edge filter and a Peltier-cooled charge-coupled device camera. The resolution of the spectrometer (grating, 1800 lines/mm) was 1 cm^–1^. The spectra were recorded in a quasi-backscattering geometry using the linearly polarized 632.817 nm line of a He/Ne gas laser. With the use of a filter, the power of the beam was adjusted to <1 mW, to protect against local heating. The spot size was 10 μm, focused by a 50× microscope objective onto the surface of the sample.

### X-ray Diffraction

For laboratory powder X-ray diffraction (PXRD), the samples were sealed into glass capillaries having an inner diameter of 0.2 mm, which were mounted on Stoe Stadi-P diffractometers in Debye–Scherrer geometry, equipped with Ge(111) monochromators and Mythen2 1K Dectris detectors. The samples were measured using Ag-K_α1_ and Cu-K_α1_ radiation with wavelengths of 0.55941 and 1.54059 Å, respectively. The structure of lithium bismuth *o*-thiophosphate (LiBiPS) was determined from powder using TOPAS 6.0.^22^ The determination of the structure involved a peak fitting and an indexing of the obtained PXRD data, followed by a Pawley fit, and the preliminary structure model (without lithium) was finally obtained using a combination of the charge flipping and simulated annealing approach.^23,24^ After identification of the heavy atoms and PS_4_ tetrahedra, the latter were restrained as rigid bodies with the freedom to rotate, stretch, and bend to a certain degree. The background of the diffraction patterns was modeled by Chebychev polynomials of the tenth order and the peak profile by using the fundamental parameter approach implemented in TOPAS.^25,26^ The instrumental resolution function had been determined by a LeBail fit^27^ of a LaB_6_ or Si standard measurement prior to the experiments.

### Neutron Powder Diffraction

Neutron powder diffraction (NPD) was performed at Spallation Neutron Source Powgen BL-11A of Oak Ridge National Laboratory. The measurements were conducted in a vanadium can having a diameter of 6 mm at 300 K using wavelengths of 0.8 and 2.665 Å. The refinement of the NPD data was performed with the use of a TOPAS template provided by Oak Ridge National Laboratory and as described by Dinnebier et al.^28^ Here, the preliminary structure model obtained from PXRD could be refined without the use of rigid bodies and including lithium positions. The obtained crystallographic data have been deposited at the Cambridge Crystallographic Data Centre (CCDC) with deposition number 2235620.

### Pair Distribution Function Analysis

Pair distribution function (PDF) analysis^29−31^ was performed on neutron [neutron pair distribution function (nPDF)] and X-ray [X-ray pair distribution function (xPDF)] data. For nPDF analysis, the data sets collected at BL-11A were normalized and merged by averaging over a range of *Q* from 4.57 to 5.33 Å^–1^, where resolution differences were minimal, to give a high resolution at a lower *Q* and a high *Q*_max_. The reduced structure function *F*(*Q*) was obtained using PDFgetN3^32^ and further Fourier transformed using a Lorch function^33^ over a *Q*_range_ from 0.5 to 30.0 Å^–1^. The data for xPDF analysis were collected using a Stoe Stadi-P diffractometer with Ag-K_α1_ radiation and 0.2 mm inner diameter glass capillary. Measurements collected at room temperature using a single Mythen2 1K Dectris detector over angular ranges of 0.405–125.145° (150 s/step), 40.5–125.145° (150 s/step), and 81.0–125.145° (300 s/step) were averaged and corrected for sample offset. An empty glass capillary was also measured and subtracted as a background. The xPDF data were further processed using PDFgetX3^34^ within the xPDFsuite^35^ and Fourier transformed using a *Q*_range_ from 0.4 to 13.0 Å^–1^. An additional xPDF measurement was performed at 100 K using an Oxford Cryostream.

Structural co-refinements to the xPDF and nPDF data were carried out using PDFgui^36^ to refine lattice parameters *a*, *b*, *c*, and β, isotropic atomic displacement parameters (ADPs) for Bi, P, and S, *U*_iso_ fixed at 0.01 Å^2^ for Li, and site positions by *C*2/*c* symmetry (with low occupancy, disordered Li sites removed). A linear peak sharpening term δ_1_^37^ was used to describe the effects of correlated motion at short distances, and the *Q*_damp_ parameter was refined for each data set to describe the effects of instrumental broadening.^38,39^

### Solid-State Nuclear Magnetic Resonance Spectroscopy

All solid-state nuclear magnetic resonance (ssNMR) measurements were performed on a Bruker Avance-III wide bore spectrometer in a magnetic field of 9.4 T. ^7^Li (Larmor frequency of 155.5 MHz), ^6^Li (Larmor frequency of 58.9 MHz), and ^31^P (Larmor frequency of 161.9 MHz) magic angle spinning (MAS) nuclear magnetic resonance (NMR) spectra were recorded in 4 mm ZrO_2_ rotors using a Bruker BL4 MAS probe at a spinning speed of 10 kHz. Due to the high reactivity of the studied materials, they were flame sealed in pyrex MAS inserts for Bruker 4 mm rotors (Wilmad Glass, product DWGSK2576-1). Measurements on all ^6^Li, ^7^Li, and ^31^P were performed using a simple Bloch Decay excitation scheme with a total of 512–4096 accumulations in each experiment. The relaxation delays were ensured to be sufficiently long to provide for complete relaxation of magnetization and ensure quantitative measurements. The spectra were referenced to the external signals of 85% H_3_PO_4_ (^31^P) and a 1 M solution of LiCl (^6^Li and ^7^Li).^40^^7^Li PFG-NMR diffusion measurements were conducted on a Bruker Avance-III 400 instrument equipped with a diff60 gradient probe (maximum gradient of 2900 G cm^–1^). Data were acquired using a stimulated echo sequence with diffusion times in the range of 10–200 ms and effective gradient durations of 1–3 ms. The measured echo-signal attenuation peaks were phase corrected, and the integrated areas were used to extract the diffusivities.

### Electrochemical Measurements

The samples were ground, and 100–250 mg was pressed using a pressure of ∼0.5 GPa into pellets with a diameter of 5 mm and having a thickness of 1–3 mm. Then the pellets were contacted with stainless steel electrodes in a TSC Battery cell of RHD Instruments. The contact pressure during the measurements was applied with the use of a spring having a certain spring constant and, thus, applying pressures of 10 MPa. For EIS, the samples were measured with a Novocontrol Technologies NEISYS potentiostat, using amplitudes of 100 and 300 mV in a frequency range from 10^6^  to 10 Hz between −10 and 60 °C within an argon-filled glovebox. To exclude contact-related issues of the measurements, a platinum film of ∼200 nm (Quorum Q150GB) was sputtered on some pellets before contact with stainless steel electrodes. For the low-temperature EIS, a pellet with a diameter of 8 mm, a thickness of 2.304 mm, and a mass of 328.3 mg was contacted with stainless steel electrodes in a custom-made Swagelok cell. Both the pressure for fabricating the pellet and the contact pressure during the measurement were adjusted similar to the values used for the high-temperature EIS measurements. The cell was placed on a copper table in a small box, which was filled with dry ice and liquid nitrogen for cooling and connected to the potentiostat. After the sample reached thermal equilibrium at −76 °C, EIS was measured using an amplitude of 100 mV in a frequency range from 10^6^ to 10^–1^ Hz. Due to systematic errors, the data points at 49.53 and 52.99 Hz have been excluded from fitting. For direct current (dc) polarization measurements, a pellet with a diameter of 8 mm was prepared and mounted in a manner similar to that used for EIS in the custom-made Swagelok cell and measured by subsequently applying potentials from 0 to 1 V in steps of 200 mV for 3 h and collecting a data point every 100 ms. For a more precise determination of the ionic conductivity via dc, the same pellet was measured at 3 and 4 V for 5 min collecting one data point per 10 ms and held at this potential for a further 12 h with an acquisition time of 1 s.

## Results and Discussion

### Structure Determination

In the Li–Bi–P–S system, the synthesis led to dark red compounds, showing a compositional range of Li_60–3*x*_Bi_16+*x*_(PS_4_)_36_. Only for values of *x* between 4.1 and 6.5 did the synthesized compounds contain minor impurities of Li_4_P_2_S_6_ of ∼1 wt %. An increase or decrease in bismuth or lithium content led to either the precipitation of BiPS_4_ or an increased fraction of an amorphous side phase, which formed Li_4_P_2_S_6_ upon heating.

**Figure 1:**
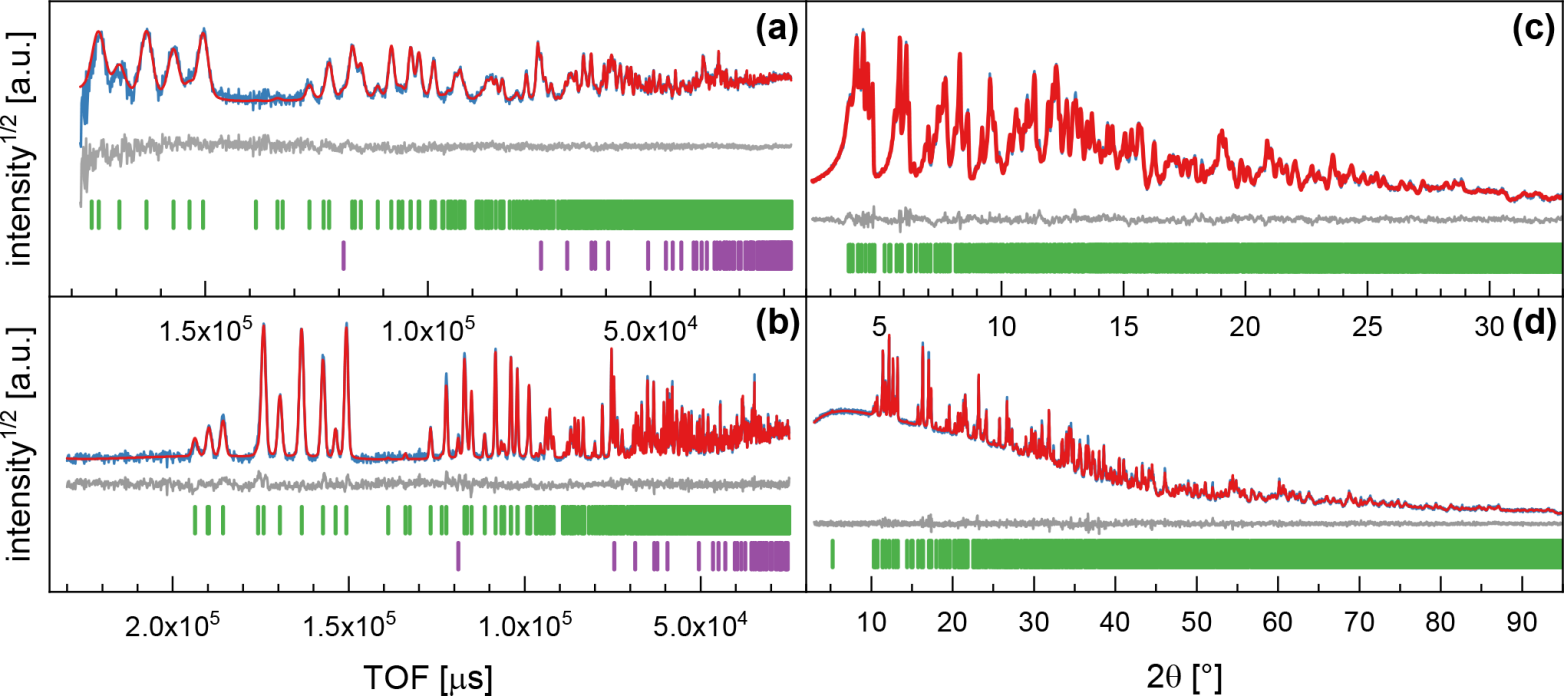
Rietveld refinements of Li_44.4_Bi_21.2_(PS_4_)_36_ for NPD data at 300 K using wavelengths of (a) 0.8 and (b) 2.665 Å and for PXRD data at room temperature using (c) Ag-K_α1_ and (d) Cu-K_α1_ radiation. The blue lines correspond to measured data, the red lines to simulated data, and the gray lines to their difference. The green ticks indicate the reflections of the main phase, whereas the purple ticks belong to minor impurities of Li_4_P_2_S_6_ (∼1 wt %) visible in only NPD.

Structure solution and characterization of LiBiPS drew on the use of several complementary techniques. While only the lattice parameters and a preliminary structure model could be identified on the basis of PXRD and electron diffraction (ED) (Figure S1), a complete structure model could be developed with the use of NPD and was verified with PDF analysis and the simulation of ED images (Figure S2). Exemplary Rietveld refinements of the PXRD and NPD data are given for Li_44.4_Bi_21.2_(PS_4_)_36_ in Figure 1 and Figure S3, and the crystallographic data are summarized in Table S1. Li_60–3*x*_Bi_16+*x*_(PS_4_)_36_ possesses a large monoclinic unit cell with space group *C*2/*c* (No. 15), and the variation of the lattice parameters with the bismuth content is shown in Figure S4. The lattice parameters for Li_60–3*x*_Bi_16+*x*_(PS_4_)_36_ with values of *x* between 4.1 and 6.5 range from 15.470 to 15.494 Å, from 10.337 to 10.271 Å, from 33.753 to 33.819 Å, and from 85.400° to 85.364° for *a*, *b*, *c*, and β, respectively. While *a* and *c* increase with *x*, *b*, β, and *V* decrease. Considering that for a replacement of one Bi^3+^ three Li^+^ need to be incorporated into the structure to maintain charge balance, this observation seems reasonable. The structure contains three bismuth, four phosphorus, 18 sulfur, and ≥10 lithium sites, all residing on general 8*f* positions and one phosphorus site at a 4*e* position (see Table S2).

The different lithium sites cannot be differentiated from one another in ^6^Li- and ^7^Li-ssNMR (see Figure S15a,b), where just one strong, asymmetric signal at −0.1  and 0.1 ppm and minor signals at 0.7 and 0.8 ppm, respectively, could be observed. In the ^31^P-ssNMR spectra depicted in Figure S15c, at least four overlapping signals at 83.7, 80.6, 77.7, and 73.7 ppm and two weak signals at 108.8 and 90.6 ppm were found. According to the determined structure of Li_44.7_Bi_21.1_(PS_4_)_36_ (see below), the four strong signals in the ^31^P ssNMR spectra can be assigned to the main phase, while the signal at ∼109 ppm can be assigned to a minor impurity of Li_4_P_2_S_6_^41^ of ∼1 wt %. The remaining signal at ∼90.6 ppm with a fractional intensity of <1% may be ascribed to Li_2_S–P_2_S_5_^42^ glass, glassy Li_4_P_2_S_7_,^43^ or amorphous BiPS_4_.

Phosphorus is found as regular -tetrahedra with P–S bond lengths in the range of 2.014–2.070 Å (see Table S3), and their existence within the structure could further be verified using Raman spectroscopy (see Figure S16). Here, the characteristic symmetric S–P–S stretching vibration of the PS_4_ tetrahedra^44^ at 413 cm^–1^ as well as the asymmetric stretching vibrations at ∼549 cm^–1^ could be identified.

**Figure 2:**
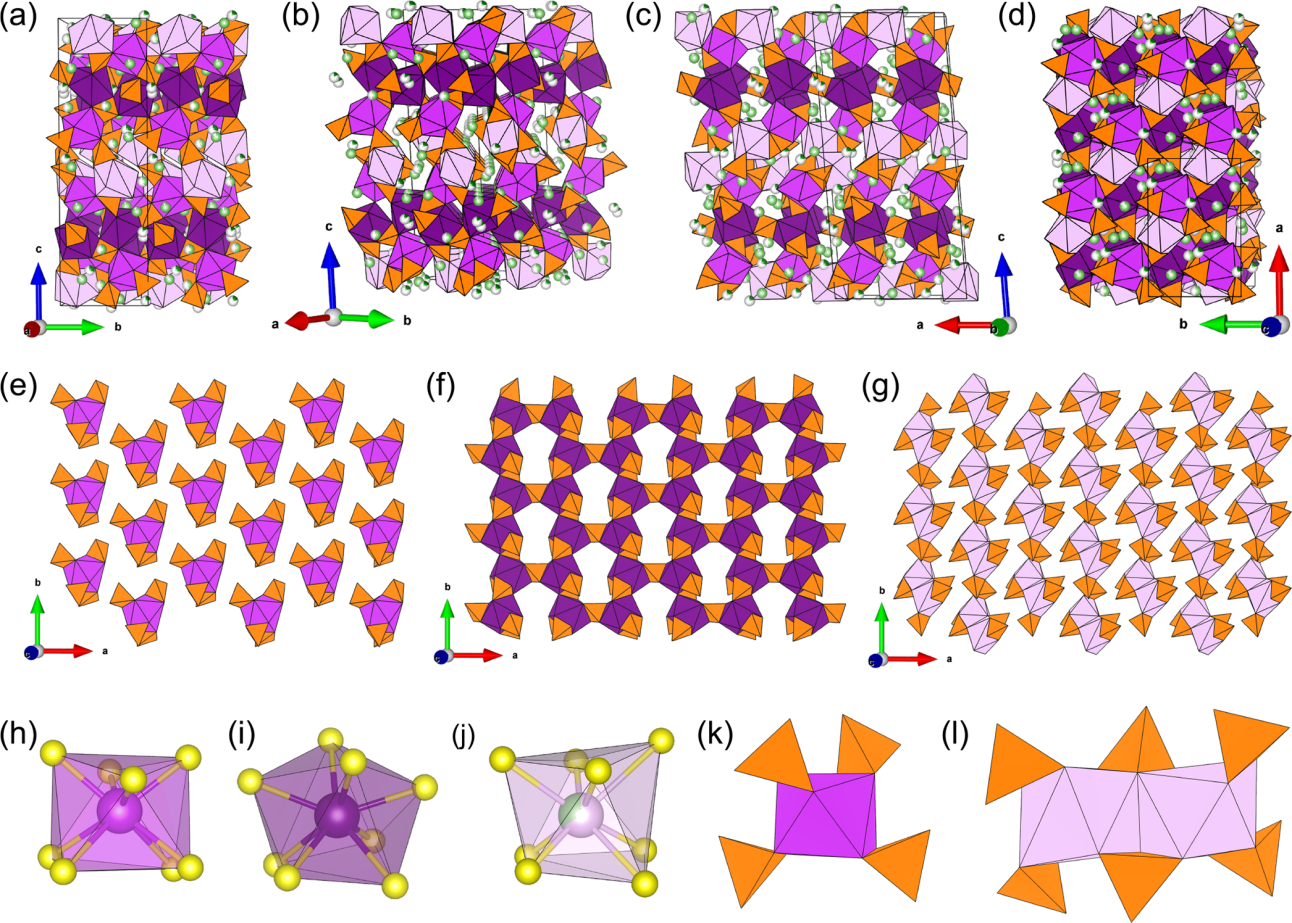
(a)–(d) Structure of Li_44.4_Bi_21.2_(PS_4_)_36_ at different orientations. Layers formed by the BiS_8_- and PS_4_-polyhedra of (e) Bi1-, (f) Bi2-, and (g) Bi3-polyhedra. Coordination environments of (h) Bi1, (i) Bi2, and (j) Bi3 and connection of (k) the BiS_8_-polyhedra of Bi1 and Bi2 with the PS_4_-tetrahedra and (l) the Bi3-polyhedra. The phosphorus tetrahedra are colored orange. The different bismuth polyhedra are colored magenta, violet, and lavender for Bi1–Bi3, respectively. The lithium ions are coloerd green.

Bismuth is coordinated by eight sulfide anions and forms distorted bicapped trigonal prisms, most likely induced by the stereochemically directing lone pair of Bi^III^ (see Figure 2h–j) as observed in other compounds.^12,13,15,44^ The Bi–S bond distances range from 2.704 to 3.516 Å (see Table S3). While bismuth sites Bi1 and Bi2 show full occupation, Bi3 sites seem to be shared with lithium ions, showing site occupation factors (SOFs) of 0.65 and 0.35, respectively, thus leading to 21.18 bismuth ions per unit cell.

According to the refinement of the NPD data, none of the remaining lithium sites show full occupation, reflecting its disordered nature. The bicapped trigonal prisms formed by Bi1 are on one hand connected via edge-sharing -tetrahedra and on the other via common corners to the Bi2- and Bi3-polyhedra. While in Li_44.4_Bi_21.2_(PS_4_)_36_ the distorted bicapped Bi1- and Bi2-prisms are directly connected to four -tetrahedra via common edges (Figure 2k), two edge-sharing Bi3-polyhedra are connected to six  tetrahedra, whereas two of them are bridging the two Bi3 polyhedra (Figure 2l). The Bi1 polyhedra are isolated from one another (Figure 2e), connected to Bi3 polyhedra by common edges and corners, and bridged by edge-sharing -tetrahedra. The Bi2-polyhedra are linked among each other via edge-sharing -tetrahedra along *a* and via common corners and bridging -tetrahedra along *b* and thus form a net-like layer in the *a–b* plane (Figure 2f). The host structure can be described as a stacking of the layers formed by the different bismuth polyhedra shown in Figure 2e–g in an ABCB-like fashion, which is illustrated in Figure 2. The lithium ions can be found within the interstices of this host framework without showing a regular coordination environment.

LiBiPS shows a structural relationship to lithium rare earth metal thiophosphates such as Li_6_Ln_3_(PS_4_)_5_ with Ln = Gd, Dy, Y, Yb, or Lu^45,46^ and Li_9_Ln_2_(PS_4_)_5_ with Ln = Nd,^47^ Ho,^48^ or Yb.^46^ Indeed, the title compound shows similar coordination environments for lithium, phosphorus, and the trivalent metal compared to Li_6_Ln_3_(PS_4_)_5_ and Li_9_Ln_2_(PS_4_)_5_, but due to the increased size of the bismuth cation as well as its stereochemically active lone pair, the arrangement slightly differs and can be seen as a structural fusion of the two different structure types of Li_6_Ln_3_(PS_4_)_5_ and Li_9_Ln_2_(PS_4_)_5_. A comparison of the PXRD patterns and the crystal structures are shown in Figures S7 and S8, respectively.

To verify the obtained structure and gain further insights into the local atomic environments, an analysis of the PDFs from neutron (nPDF) and X-ray (xPDF) total scattering data was performed. As with the diffraction analysis, differences in the weighting of the partial contributions to the total PDF for neutrons versus X-rays, as shown in Figure S9, also provide complementary sensitivities to different partial contributions to the structure.^31^ The associated *F*(*Q*) functions are listed in Figure S14.

Structure-independent peak fitting indicates a strong Gaussian distribution of the P–S bond lengths with a mean value of 2.0427(14) Å determined between the nPDF and xPDF data. The Bi–S distance distribution is more complex due to the various local environments, which may be explained by the stereochemically active lone pair of Bi^III^ as observed in other compounds,^12,13,15,44^ but shows a maximum at ∼2.840(8) Å. A negative peak observed in the nPDF data at 2.450(4) Å, due to the negative neutron scattering length of ^7^Li, can be assigned to neighboring Li–S distances.

Analysis of the local structure was performed using the crystal structure model determined above. Comparison of the simulated PDF from the as-determined structure without modification to the ADPs indicates significantly increased local correlations (i.e., sharper pair distance distributions) between P–S, Li–S, Bi–S, and S–S nearest neighbor pairs that are unaccounted for in the Rietveld refinements (see Figure S10). A refinement of only the lattice parameters, thermal parameters, and a correction term for correlated motion δ_1_ still shows misfit over short distances, primarily in the range of 2.25–5.0 Å. Further refinement of the site positions gives a highly satisfactory fit and suggests the presence of local structuring between neighboring polyhedra that deviates slightly from the average structure, particularly with respect to the sulfur substructure. See Tables S4 and S5 for refinement details and Figure S11 for a comparison of structures. All partial contributions before and after refinement of the site positions are provided in Figure S12.

**Figure 3:**
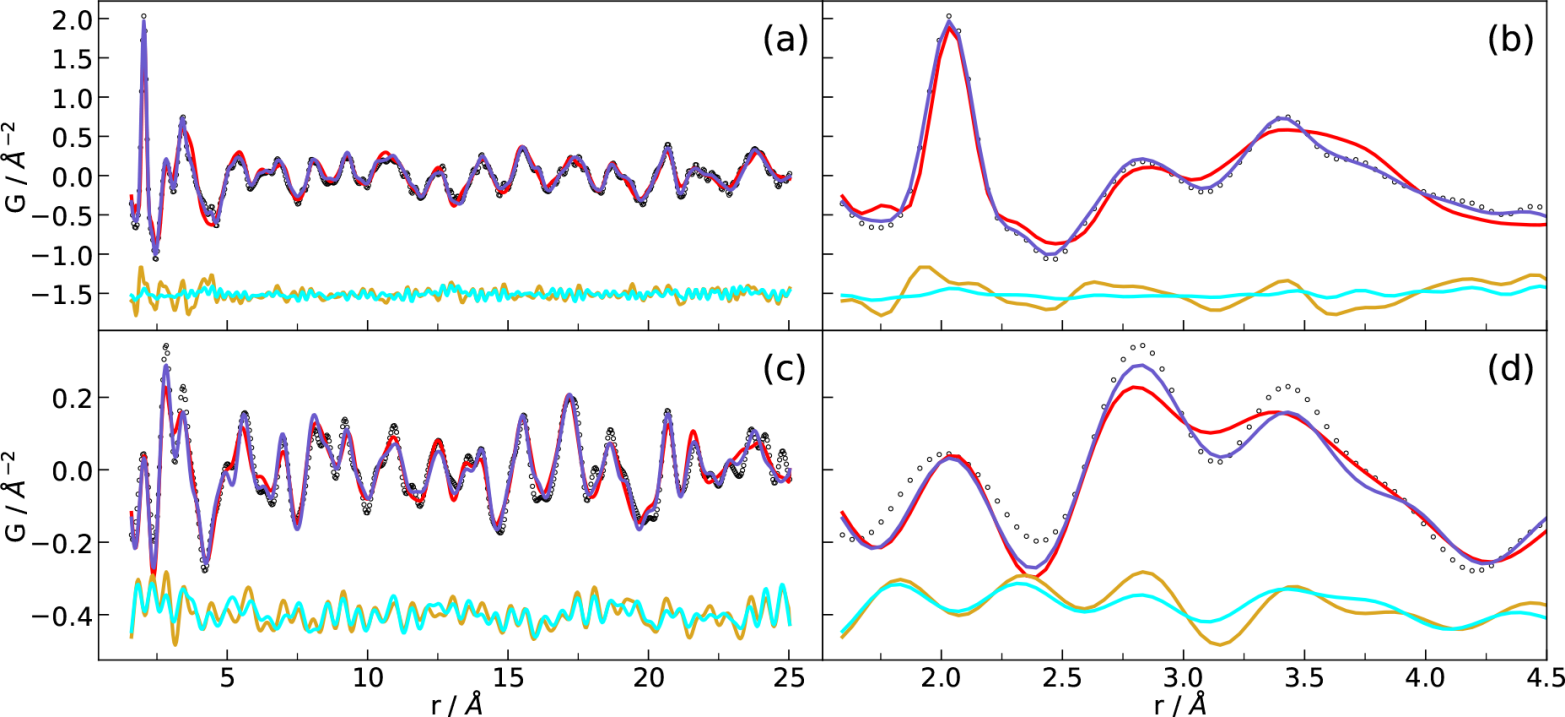
PDF analysis from (a and b) neutron data and (c and d) X-ray data for Li_44.4_Bi_21.2_(PS_4_)_36_ compared to the simulations resulting from co-refinements. Measured data (empty circles), simulation from the structure as determined from NPD with only lattice parameters, ADPs, and δ_1_ refined (red), and simulation with site positions refined (blue). The yellow and cyan lines correspond to the differences in the structure models obtained from the former and latter refinements, respectively.

Assessment of the resulting Li–S partial PDF (Figure S13) suggests a roughly bimodal distance distribution with primary maxima at approximately 2.52 and 2.83 Å. This is in agreement with the Li–S partials for the related published structures of Li_9_Ln_2_(PS_4_)_5_, also showing a strong bimodal distribution, and Li_6_Ln_3_(PS_4_)_5_, showing a slightly more continuous distribution. LiBiPS appears to show a relatively larger population of Li at a longer coordination distance. More Li disorder in LiBiPS may be driven by the greater difficulty in forming optimal coordination environments in this system. As mentioned above, the different lithium sites could not be differentiated in ssNMR, and thus, the different contributions cannot be assigned to specific sites. However, the results do suggest that the lower occupation Li sites that appear with shortened Li–S bonds (≲2.3 Å) are likely a result of the representation of disorder in the average crystallographic density but not representative of the instantaneous local environments. We do not detect any distinct negative peaks at these shorter distances. Overall, the fits resulting from the refinements reaffirm the model obtained by the crystal structure solution and are shown in Figure 3.

### Lithium Ion Conductivity and Diffusion Pathways

**Figure 4:**
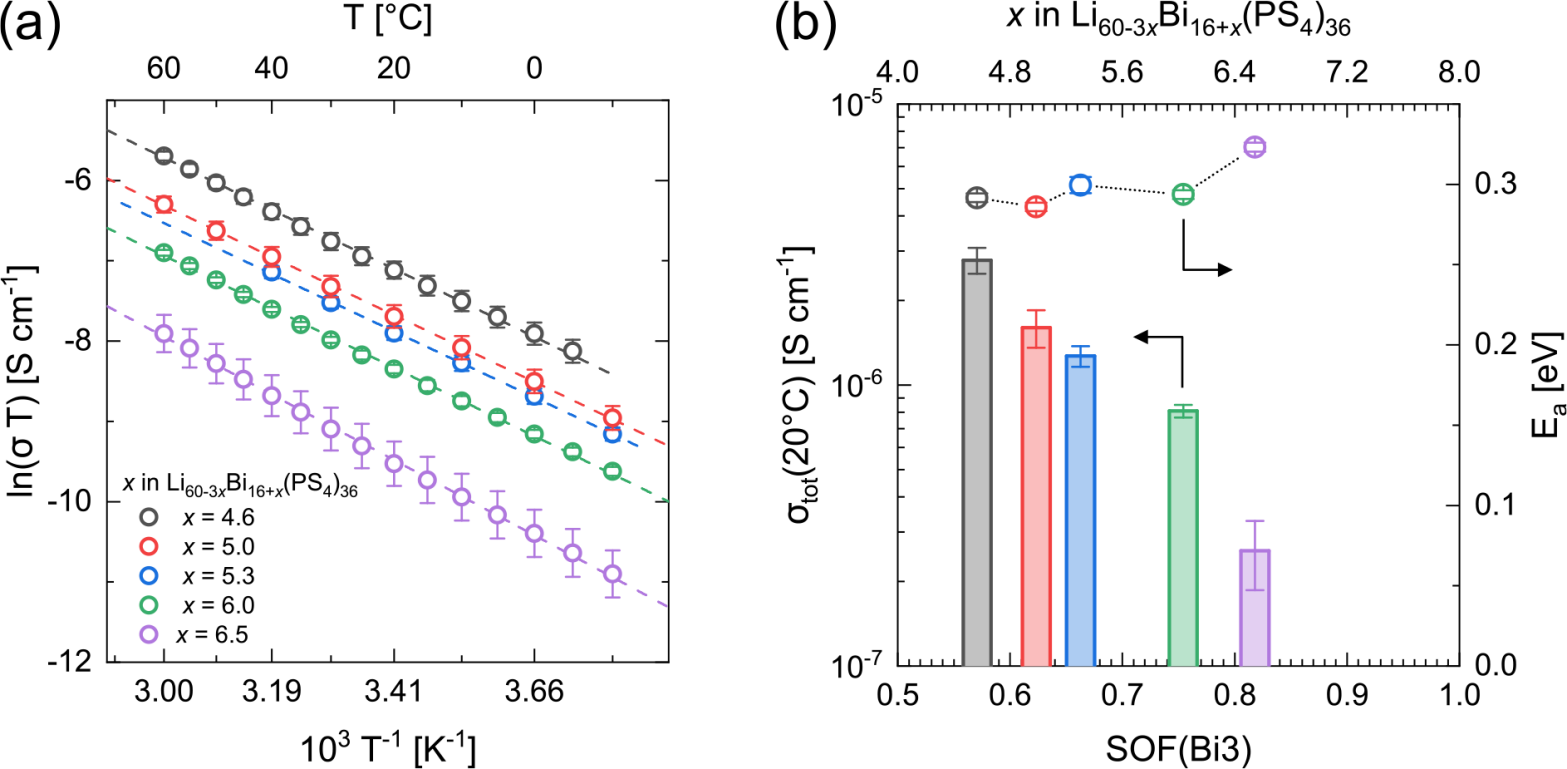
Results of the EIS measurements. (a) Arrhenius graphs of the different Li_60–3*x*_Bi_16+*x*_(PS_4_)_36_ species with their total ionic conductivities at 20 °C and (b) activation energies as a function of the SOF of Bi3 and of bismuth content *x*.

Structures with high levels of site disorder and low local coordination symmetry often show good ionic transport properties.^49,50^ We thus studied the lithium ion conductivity with the use of EIS. A discussion of the equivalent circuit models (ECMs) used for fitting the impedance data (Figure S17) as well as the obtained Nyquist and Bode plots (Figures S18–S21) and the respective values of the different equivalent circuit elements (ECEs) at a given temperature (Tables S6 and S7) can be be found in the Supporting Information, whereas the obtained Arrhenius graphs, ionic conductivities at 20 °C, and activation energies for ion diffusion are given in Figure 4. Li_45_Bi_21_(PS_4_)_36_ exhibits a total ionic conductivity of 1.6 × 10^–6^ S cm^–1^ at 20 °C with an activation energy of 0.29 eV. Increasing the bismuth content and accordingly decreasing the lithium content on the mixed Bi/Li sites appear to continuously decrease the ionic conductivity at 20 °C from 2.8 × 10^–6^ to 2.6 × 10^–7^ S cm^–1^. This may be ascribed to either a decreased number of available lithium ions as charge carriers or a further restriction of the diffusion pathways (narrowing or blocking) when the bismuth content is increased. The activation energy appears not to show a trend that is as pronounced, but overall behaves similarly and increases with bismuth content from 0.29 to 0.32 eV.

The lithium ion diffusion in Li_44.4_Bi_21.2_(PS_4_)_36_ has been additionally investigated with the use of ^7^Li-PFG-NMR spectroscopy, and the results are summarized in Figure S23. At 30 °C, *T*_1_ and *T*_2_ relaxation times were 6.04  and 5.99 × 10^–4^ s, respectively, leading to a tracer diffusion coefficient *D*_tr_^NMR^ of 1.1 × 10^–14^ m^2^ s^–1^. An activation energy for lithium ion diffusion *E*_a_^NMR^ of 0.40 eV was obtained from the PFG-NMR measurements. In contrast, the ^7^Li spin–lattice as well as spin–spin relaxation rates, which are typically associated with short-range (i.e., site-to-site) Li hopping processes, showed activation energies of 0.19 and 0.17 eV (see Figure S23a). These are comparable to other sulfide-based lithium ion conductors.^51−54^ The average diffusion length estimated from PFG-NMR measurements according to eq S12 is 661 Å at 30 °C and thus points to predominantly intracrystalline/bulk diffusion processes within the time frame of the measurement, whereas EIS accounts for intra- and intercrystalline ionic motion. The diffusion rate of an elementary jump was approximated according to eq S13. For a jump distance of 2.219 Å [obtained from *softBV* calculations (see below)], a jump rate of 1.33 × 10^6^ s^–1^ was obtained. According to the Nernst–Einstein relationship given in eq S14, this results in an ionic conductivity σ_ion_^NMR^ of 5.5 × 10^–6^ S cm^–1^ at 30 °C (compared to a σ_ion_^EIS^ of 1.8 × 10^–6^ S cm^–1^), when all lithium ions are assumed to be mobile (44.4 lithium ions per unit cell volume ≡ 8.242 × 10^27^ m^–3^) and with a Haven ratio of unity. The obtained values are only approximations because the real number of active charge carriers is unknown and the Haven ratio^55^ as well as the Bardeen–Herring tracer correlation factor^56^ can deviate from unity.

The electronic conductivity was estimated using chronoamperometric dc polarization measurements, as shown in Figure S22a. The equilibrium currents after successive potential steps of 200 mV showed an ohmic behavior as depicted in Figure S22b. Here, an electrical resistance *R*_el_ in the range from 5.7 × 10^8^ to 8.7 × 10^8^ Ω was observed for Li_44.4_Bi_21.2_(PS_4_)_36_, leading to electronic conductivities σ_el_^dc^ in the range from 3.4 × 10^–10^ to 5.2 × 10^–10^ S cm^–1^ at 20 °C . According to eq S9, the ionic transference number (*t*_ion_) was estimated to be 0.999 (from a σ_ion_^EIS^ of 5.2 × 10^–7^ S cm^–1^ at 20 °C using the same pellet). The ionic conductivity from dc polarization measurements (σ_ion_^dc^) can be obtained from the initial currents *I*_init_ of the polarization^57,58^ according to eqs S7 and S8. Because *I*_init_ strongly depends on the acquisition time of the measurement, an additional fast dc polarization measurement with an acquisition time of 10 ms was used for a more precise determination of *I*_init_ (see eqs S7 and S8). Here, ionic conductivities σ_ion_^dc^ of ∼3.8 × 10^–7^ and ∼4.8 × 10^–7^ S cm^–1^ were observed for 3 and 4 V, respectively, which is in good agreement with the measured total ionic conductivity obtained from EIS (σ_ion_^EIS^ = 5.2 × 10^–7^ S cm^–1^). The estimation of the conductivity diffusion coefficient *D*_σ_^dc^ via the Nernst–Einstein approximation given in eq S10 is ambiguous as long as the real number of charge carriers is unknown. Attempts to determine the charge carrier concentration with the use of transient ionic current (TIC)^59,60^ measurements were not successful.

**Figure 5:**
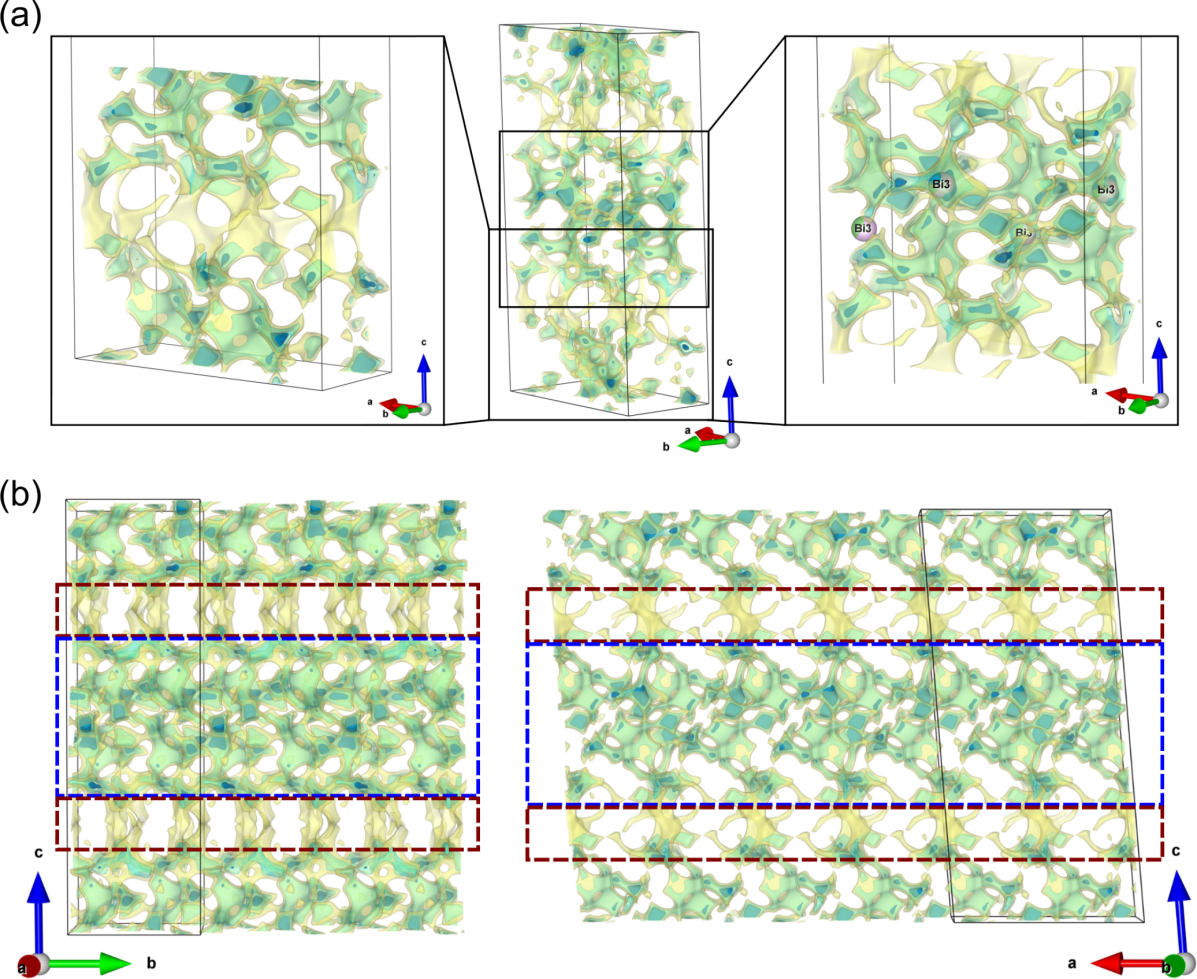
BVEL of Li_44.4_Bi_21.2_(PS_4_)_36_ at different energy levels. (a) Overview of the BVEL within the unit cell. The magnifications of certain regions are provided to highlight some of the diffusion pathways. In the right panel, the mixed occupied Bi3/Li3 site is also shown, whereas all other atoms have been omitted. (b) BVEL for three unit cells along different orientations. The blue and red boxes highlight regions of high and low diffusion pathway densities, respectively. The BVEL is colored blue for low energies (−2.75 eV), and higher energies are colored green (−2.50 eV) and yellow (−2.25 eV).

To understand the origin of the unexpectedly low lithium ion conductivity, in spite of the high level of disorder, we investigated the Li ion percolation pathways using the *softBV*(61−63) approach. The obtained bond valence energy landscape (BVEL), the respective site energies along the most dominant diffusion trajectories, and the resulting energy barriers along these paths are shown in Figure 5 and Figure S24, respectively. Unlike the fast ion conductor Li_10_GeP_2_S_12_ (>10^–2^ S cm^–1^), which shows an extended quasi-three-dimensional network of lithium diffusion pathways at room temperature,^64−66^ the lithium ion percolation pathways in LiBiPS seem to extend predominantly in a helical pseudo-two-dimensional fashion (one-dimensional helical paths, which show a branching to two dimensions, but this is associated with higher energies or narrowing of the pathways) within the *a–b* plane. Therefore, layers with a higher density of low-energy pathways are observed (see the red and blue boxes in Figure 5b), which tend to be disconnected from each other along *c*. The different lithium percolation pathways in LiBiPS show energy barriers from 0.10 to 0.48 eV in the jump distance region of 1.6–3.8 Å as illustrated in Figure S24b. The restricted dimensionality of the diffusion pathways and their narrow/bottleneck-like nature are the reasons for the overall low ionic conductivities. For the related lanthanide compounds Li_9_Ho_2_(PS_4_)_5_ and Li_15_Ho_7_(PS_4_)_12_,^48^ one-dimensional pathways with energy barriers of 0.378 and 0.727 eV, respectively, and three-dimensional pathways with energy barriers of 0.471 and 0.838 eV, respectively, have been reported. Notably, in LiBiPS, the Bi3/Li3 sites are also part of the lithium ion percolation pathways (see the right side of Figure 5a) in the high-density region (blue box in Figure 5b) and a variation of the bismuth content thus alters the diffusion pathways along these sites. An increase in the bismuth content at these sites is expected to lead to a reduction in the number of opportunities for diffusion due to a blocking of diffusion pathways, thus resulting in a decrease in ionic conductivity as observed in EIS.

## Conclusion

With this work, we present the synthesis and in-depth characterization of the first lithium-containing bismuth *o*-thiophosphate Li_60–3*x*_Bi_16+*x*_(PS_4_)_36_ with a compositional range of *x* from 4.1 to 6.5. The structure of LiBiPSs has been comprehensively examined with the use of PXRD, NPD, ED, xPDF and nPDF analysis, ssNMR, PFG-NMR spectroscopy, and Raman spectroscopy. Li_60–3*x*_Bi_16+*x*_(PS_4_)_36_ compounds with values of *x* between 4.1 and 6.5 possesses a complex monoclinic structure [*C*2/*c* (No. 15)] and a large unit cell with the following parameters: *a* = 15.472–15.491 Å, *b* = 10.337–10.295 Å, *c* = 33.755–33.808 Å, and β = 85.394–85.378°. A structural relationship to Li_6_Ln_3_(PS_4_)_5_ and Li_9_Ln_2_(PS_4_)_5_ is evident, and the structure of the title compound can be described as a fusion of the latter structure types. The lithium ions are randomly distributed within the interstices formed by the densely connected bismuth thiophosphate host framework. In spite of the large degree of disorder, an overall low total ionic conductivity on the order of 2.6 × 10^–7^ to 2.8 × 10^–6^ S cm^–1^ at 20 °C was observed, with activation energies for ion diffusion in the range of 0.29–0.32 eV depending on the bismuth content of Li_60–3*x*_Bi_16+*x*_(PS_4_)_36_. The low long-range diffusivity of the lithium ions [*D*_tr_^NMR^(30 °C) = 1.1 × 10^–14^ m^2^ s^–1^] can be ascribed to the predominantly one-dimensional and narrow helical diffusion pathways within the *a–b* plane. In spite of the disordered nature of the lithium ions, which generally is conducive to high ionic conductivities, the overall rather dense framework structure, paired with low-dimensional lithium diffusion pathways, gives rise to limited long-range diffusion of lithium ions.

## Supporting Information Available

Electron diffraction images and a comparison with the simulation, crystallographic information obtained from the Rietveld refinements of the NPD and PXRD data, ^6^Li-, ^7^Li-, and ^31^P-ssNMR spectra, Raman spectra, results of the *softBV* calculations, details about the electrochemical characterization like the fitting procedure, Nyquist, Bode and current versus voltage plots, and the results obtained from PFG-NMR spectroscopy (PDF)

## Footnotes

a Note that a preprint of this article was uploaded to *chemRxiv*(19) prior to publication.
