# Supplementary material for: Structure and Ionic Conductivity of Li-Disordered Bismuth o-Thiophosphate Li60–3xBi16+x(PS4)36
Source: Inorg Chem. 2023 Jun 29;62(27):10655–64. doi: 10.1021/acs.inorgchem.3c01028 (PMC10336968; doi:10.1021/acs.inorgchem.3c01028)
Supplement: Supplementary file 1 — ic3c01028_si_001.pdf [file ic3c01028_si_001.pdf]

Supporting Information

Structure and Ionic Conductivity of Li-Disordered Bismuth *o*-Thiophosphate $Li_{60-3x}Bi_{16+x}(PS_4)_{36}$

Maximilian A. Plass[†,‡], Maxwell W. Terban[†], Tanja Scholz[†], Igor Moudrakovski[†], Viola Duppel[†], Robert E. Dinnebier[†] and Bettina V. Lotsch*[†,‡]

† *Max Planck Institute for Solid State Research, Heisenbergstraße 1, 70569 Stuttgart*
‡ *University of Munich (LMU), Butenandtstraße 5-13, 81377 Munich*

E-Mail: b.lotsch@fkf.mpg.de

Electron diffraction in transmission electron microscopy

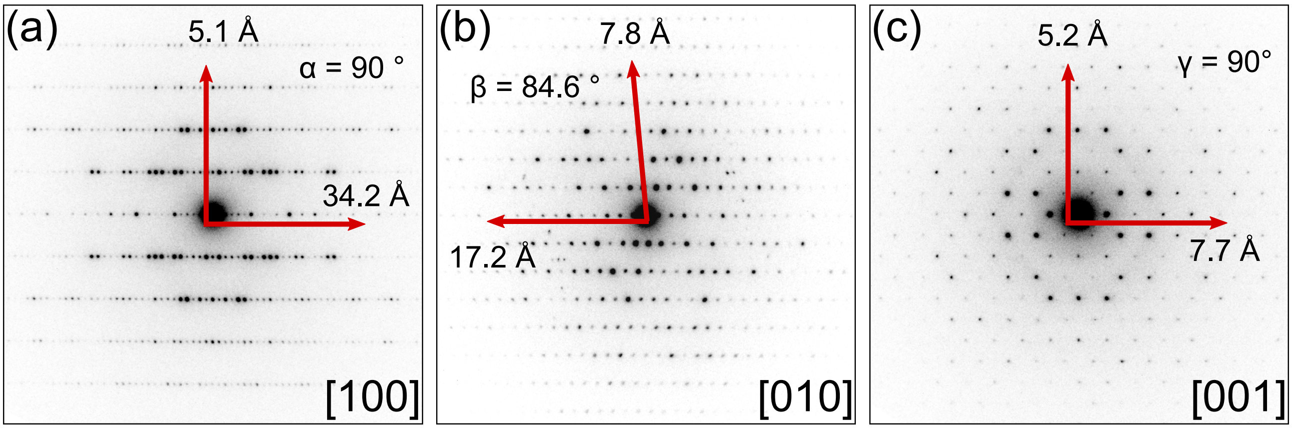

Figure S1. Precession electron diffraction images of $Li_{45.9}Bi_{20.7}(PS_4)_{36}$ along different crystallographic axes: (a) [100], (b) [010] and (c) [001].

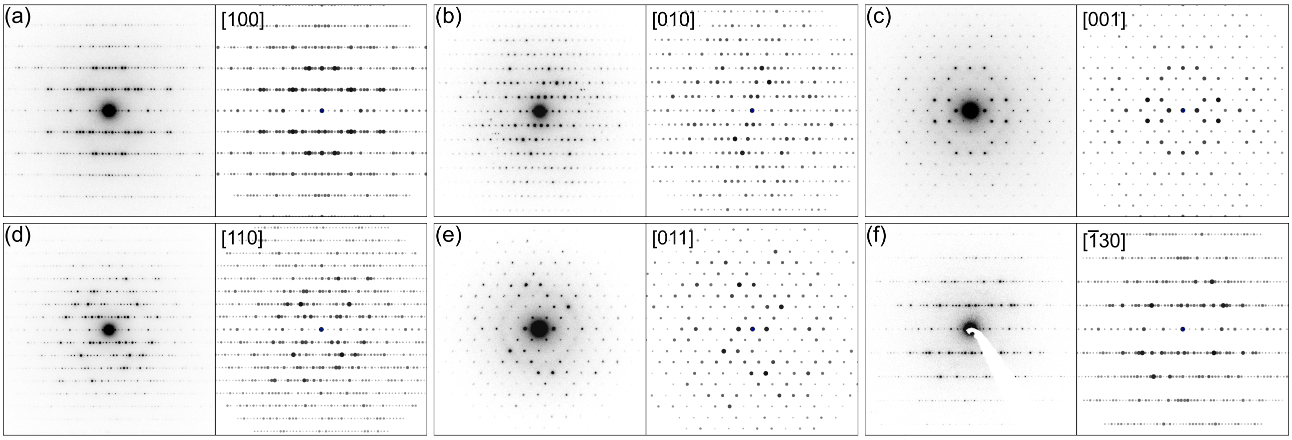

Figure S2. Comparison of measured (left) and simulated (right) precession electron diffraction images of $Li_{45.9}Bi_{20.7}(PS_4)_{36}$ at different crystallographic orientations in (a)–(f).

X-ray and neutron diffraction

Table S1. Details of the measurement conditions as well as crystallographic data obtained from Rietveld refinements of NPD and PXRD data.

source	NPD		PXRD	
			Ag-$K_{\alpha 1}$	Cu-$K_{\alpha 1}$
formula	$Li_{44.4}Bi_{21.2}(PS_4)_{36}$		$Li_{44.7}Bi_{21.1}(PS_4)_{36}$	$Li_{44.4}Bi_{21.2}(PS_4)_{36}$
refined composition	$Li_{44.14}Bi_{21.20}(PS_4)_{36}$		$Li_{44.04}Bi_{21.32}(PS_4)_{36}$	$Li_{44.04}Bi_{21.30}(PS_4)_{36}$
weight [g/mol]	10470.980		10452.164	10470.980
density [g/cm^3]	3.227(4)		3.235(3)	3.241(3)
T [K]	300		RT	RT
λ [Å]	0.8	2.665	0.55941	1.54060
crystal system	monoclinic			
space group (no.)	$C2/c$ (15)			
a [Å]	15.467(1)	15.4866(2)	15.4936(6)	15.4792(2)
b [Å]	10.3129(7)	10.3232(2)	10.3159(5)	10.3123(2)
c [Å]	33.767(2)	33.8046(5)	33.802(2)	33.7792(7)
β [°]	85.394(5)	85.395(1)	85.392(3)	85.391(1)
V [Å^3]	5369.8(7)	5386.9(1)	5385.2(4)	5374.6(2)
Z	1			
d-space [Å]	7.884–0.816	10.200–1.102	14.240–0.662	29.422–1.045
χ^2	1.624	3.246	1.513	3.827
R_p	5.492	3.907	1.572	1.927
R_{wp}	3.422	3.474	2.061	2.615

The quality factors were calculated according to Equations S1 to S4 and for a detailed explanation one should refer to Toby [1].

$$R_p = \frac{\sum |Y_{o,i} - Y_{c,i}|}{\sum |Y_{o,i}|} \tag{S1}$$

$$R_{wp} = \sqrt{\frac{\sum w_i \left(Y_{o,i} - Y_{c,i}\right)^2}{\sum w_i Y_{o,i}^2}} \tag{S2}$$

$$R_{exp} = \sqrt{\frac{M - P}{\sum w_i Y_{o,i}^2}} \tag{S3}$$

$$\chi^2 = \left(\frac{R_{wp}}{R_{exp}}\right)^2 \tag{S4}$$

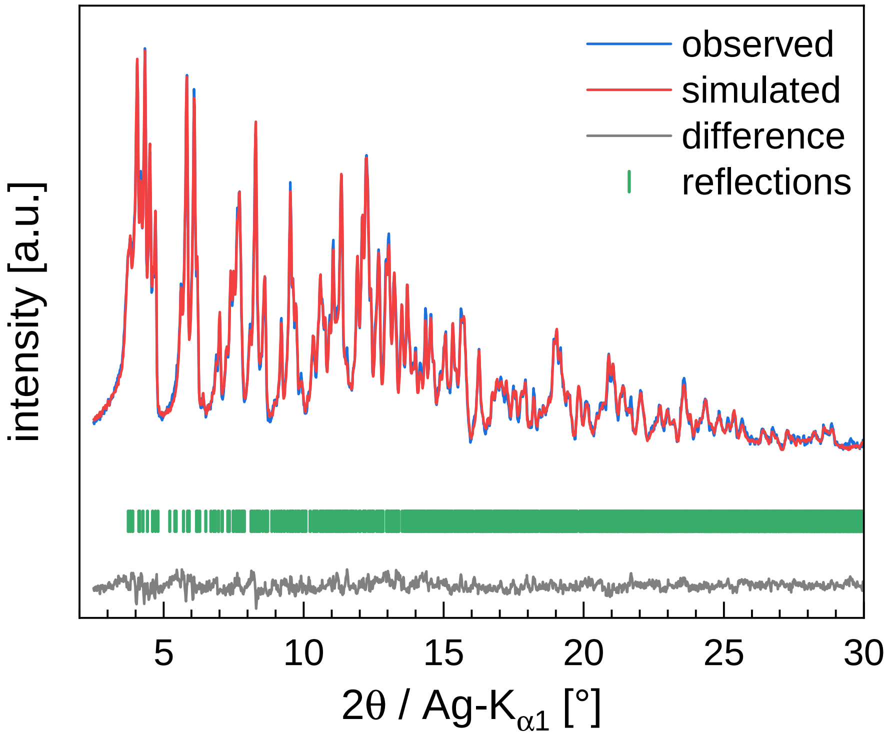

Figure S3. Rietveld refinement of PXRD data of $Li_{44.4}Bi_{21.2}(PS_4)_{36}$ at 100 K.

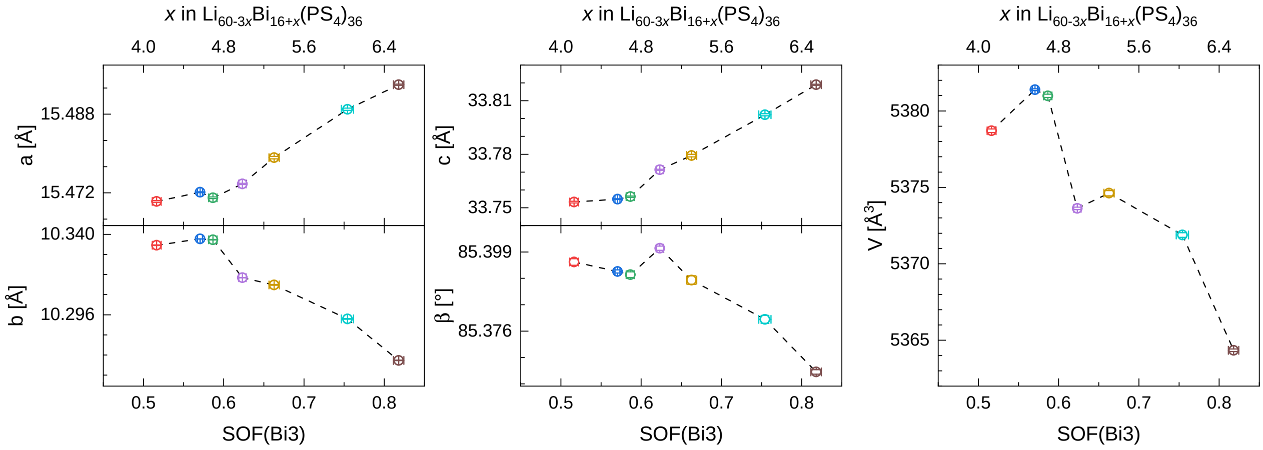

Figure S4. Variation of the lattice parameters a, b, c, β and V with the site occupation factor (SOF) of the mixed bismuth/lithium site Bi3.

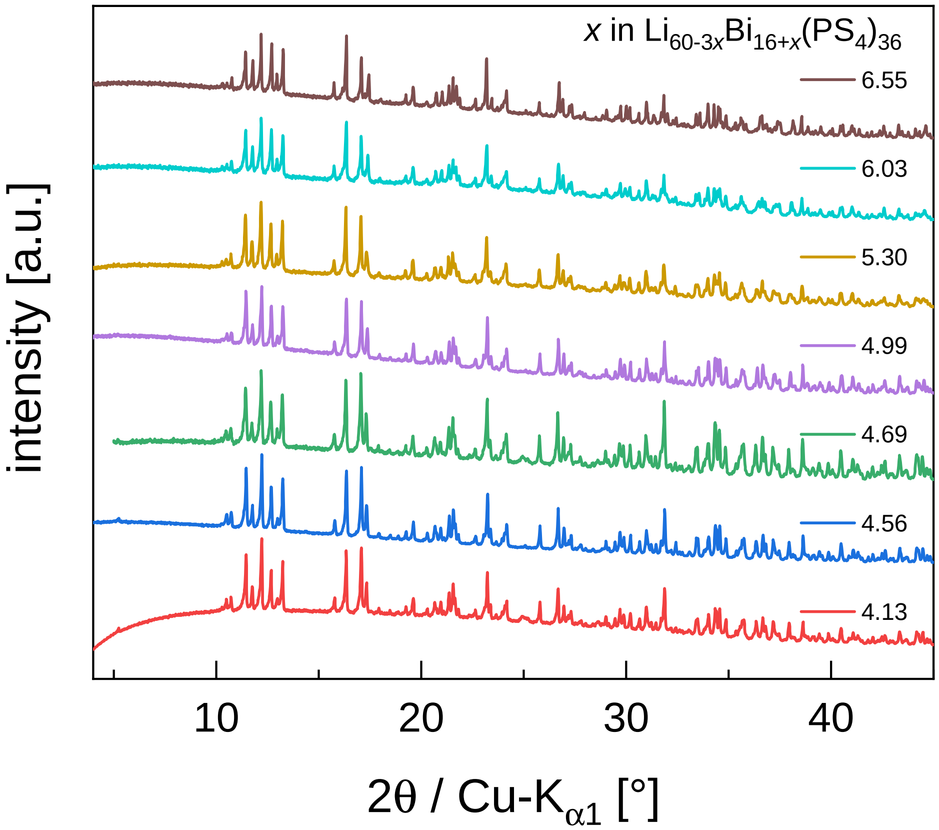

Figure S5. PXRD patterns of $Li_{60-3x}Bi_{16+x}(PS_4)_{36}$ as a function of x.

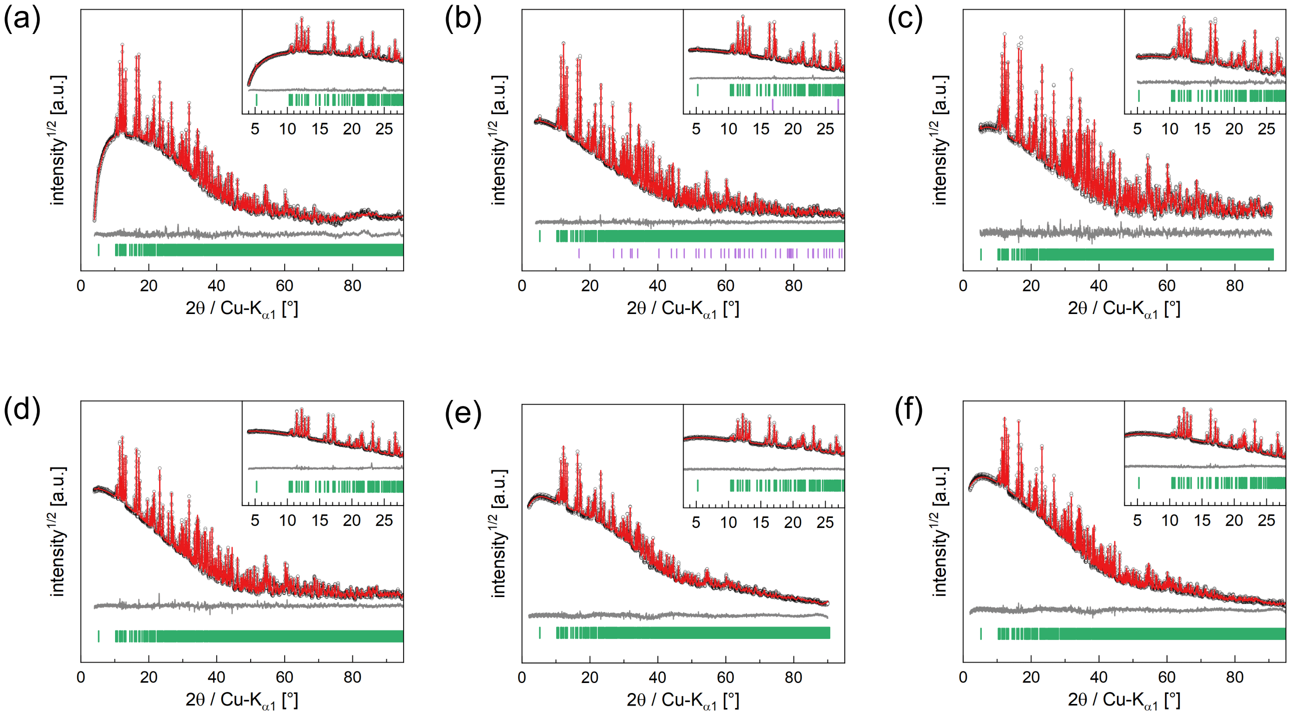

Figure S6. Rietveld refinements of different samples of $Li_{60-3x}Bi_{16+x}(PS_4)_{36}$ as a function of x: (a) 4.13, (b) 4.56, (c) 4.69, (d) 4.99, (e) 6.03 and (f) 6.55. The black open circles denote measured data, the red line corresponds to the simulation, the gray curve represents the difference between observed and simulated and the green ticks correspond to the distinct reflections. The violet ticks in (b) indicate an impurity of $Li_4P_2S_6$ with a phase fraction of < 2 wt.%.

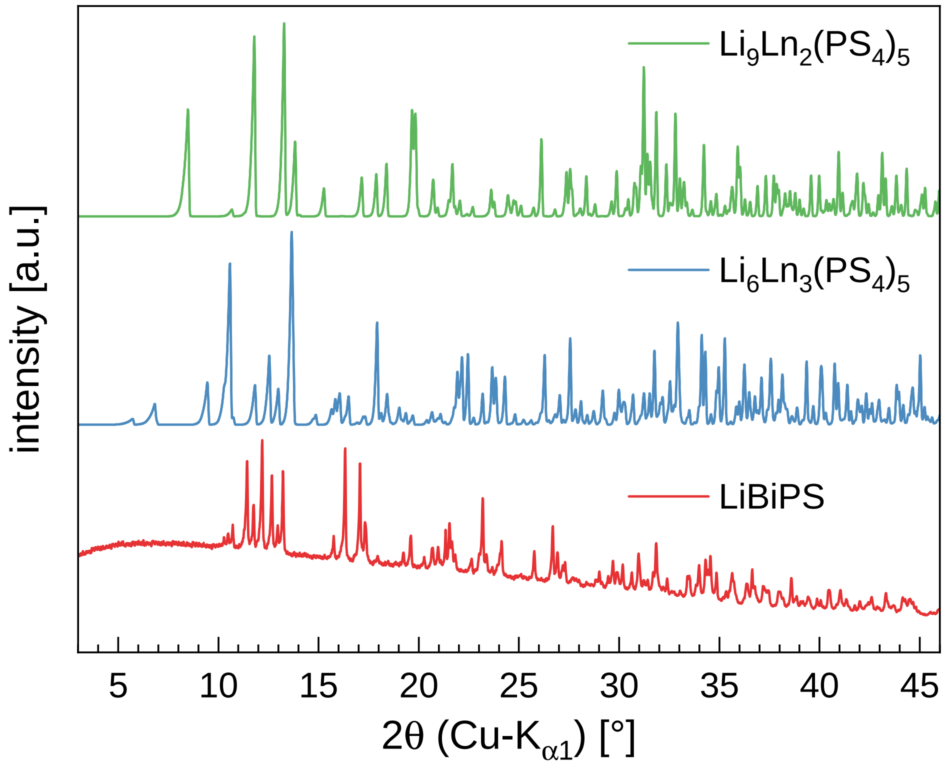

Figure S7. Comparison of the measured diffraction pattern of $Li_{44.4}Bi_{21.2}(PS_4)_{36}$ with simulated patterns of lithium lanthanide thiophosphates.

Table S2. Atomic positions and isotropic thermal displacement parameters in $Li_{44.4}Bi_{21.2}(PS_4)_{36}$.

atom	Wyckoff site	x	y	z	occupation	B_{iso}
Bi1	8f	0.69718(35)	0.74898(42)	0.88886(14)	1	2.72(13)
Bi2	8f	0.71505(30)	0.66743(41)	0.27799(12)	1	2.00(11)
Bi3	8f	0.43807(48)	0.87591(72)	0.94962(22)	0.644(7)	1.63(24)
Li3	8f	0.43807(48)	0.87591(72)	0.94962(22)	0.356(7)	1.63(24)
S1	8f	0.45990(73)	0.56999(119)	0.20565(33)	1	0.78(6)
S2	8f	0.64770(75)	0.56750(111)	0.94461(34)	1	0.78(6)
S3	8f	0.31011(72)	0.80618(111)	0.90150(35)	1	0.78(6)
S4	8f	0.73154(69)	0.34604(124)	0.54585(33)	1	0.78(6)
S5	8f	0.15064(71)	0.34033(113)	0.33442(33)	1	0.78(6)
S6	8f	0.07949(72)	0.37976(108)	0.01174(35)	1	0.78(6)
S7	8f	0.09794(76)	0.63449(108)	0.19114(34)	1	0.78(6)
S8	8f	0.44137(76)	0.60755(102)	0.96774(34)	1	0.78(6)
S9	8f	0.28274(73)	0.49543(109)	0.15935(35)	1	0.78(6)
S10	8f	0.68249(74)	0.52628(99)	0.14132(37)	1	0.78(6)
S11	8f	0.11392(73)	0.60091(102)	0.61706(33)	1	0.78(6)
S12	8f	0.66271(72)	0.85079(106)	0.80440(37)	1	0.78(6)
S13	8f	0.30919(74)	0.58444(113)	0.76765(32)	1	0.78(6)
S14	8f	0.68651(74)	0.85860(107)	0.48642(36)	1	0.78(6)
S15	8f	0.40262(75)	0.20083(101)	0.77411(34)	1	0.78(6)
S16	8f	0.00631(70)	0.85283(105)	0.91389(36)	1	0.78(6)
S17	8f	0.40186(75)	0.96314(107)	0.58394(34)	1	0.78(6)
S18	8f	0.02837(72)	0.83387(109)	0.37530(34)	1	0.78(6)
P1	8f	0.97229(42)	0.04367(69)	0.57646(20)	1	1.16(7)
P2	8f	0.33338(42)	0.05785(63)	0.35881(21)	1	1.16(7)
P3	8f	0.34089(45)	0.95923(67)	0.53190(20)	1	1.16(7)
P4	8f	0.76635(41)	0.48865(62)	0.18422(21)	1	1.16(7)
P5	4e	0	0.18262(87)	0.25000	1	1.16(7)
Li1	8f	0.06278(140)	0.19672(210)	0.15596(61)	0.93(3)	2.32(38)
Li2	8f	0.92568(132)	0.12832(194)	0.48485(59)	1.00(3)	2.32(38)
Li4	8f	0.15325(170)	0.22938(223)	0.41030(70)	0.80(3)	2.32(38)
Li5	8f	0.08837(153)	0.86809(205)	0.17117(68)	0.89(3)	2.32(38)
Li6	8f	0.02626(246)	0.47102(401)	0.30342(108)	0.50(3)	2.32(38)
Li7	8f	0.29539(341)	0.42444(482)	0.43337(153)	0.41(3)	2.32(38)
Li8	8f	0.07405(474)	0.78278(675)	0.11727(210)	0.28(3)	2.32(38)
Li9	8f	0.32102(602)	0.54298(937)	0.51286(274)	0.21(3)	2.32(38)
Li10	8f	0.47297(809)	0.44331(1160)	0.24053(367)	0.15(2)	2.32(38)

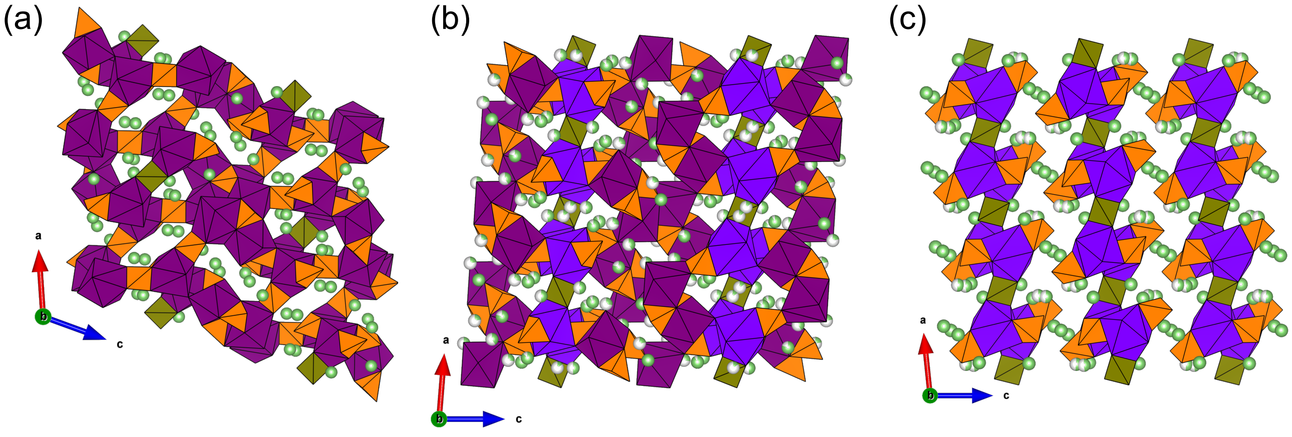

Figure S8. Comparison of the crystal structures observed for (a) $Li_6\mathit{Ln}_3(PS_4)_5$, (b) $Li_{44.4}Bi_{21.2}(PS_4)_{36}$ and (c) $Li_9\mathit{Ln}_2(PS_4)_5$. The coordination polyhedra of phosphorous are depicted in orange, whereas phosphorous occupying the 4e site is highlighted in dark yellow. The coordination polyhedra of the trivalent metals are given in different shades of purple in order to highlight the structural relationships between the different structure types. Lithium ions are given in green.

Table S3. Bond lengths in $Li_{44.4}Bi_{21.2}(PS_4)_{36}$.

atom A	atom B	bond length A–B [Å]	atom A	atom B	bond length A–B [Å]
Bi1	S2	2.720(12)	P1	S16	2.026(13)
	S4	2.733(13)		S8	2.031(13)
	S17	2.800(12)		S2	2.055(14)
	S18	2.820(12)		S18	2.061(13)
	S9	3.003(12)	P2	S3	2.022(13)
	S10	3.094(12)		S11	2.029(13)
	S12	3.125(13)		S9	2.048(14)
	S11	3.289(12)		S7	2.076(13)
Bi2	S5	2.738(12)	P3	S6	2.017(13)
	S9	2.763(12)		S14	2.033(13)
	S1	2.898(12)		S17	2.060(14)
	S13	2.951(12)		S4	2.078(13)
	S15	2.961(13)	P4	S12	2.040(13)
	S13	3.059(12)		S10	2.058(14)
	S7	3.171(13)		S5	2.063(13)
	S12	3.241(12)		S13	2.066(13)
Bi3	S3	2.755(14)	P5	S1	2.031(13)
	S14	2.783(13)		S15	2.045(12)
	S17	2.788(13)			
	S8	2.836(13)			
	S6	2.840(13)			
	S6	3.151(14)			
	S11	3.375(13)			
	S18	3.517(13)			

Pair distribution function analysis

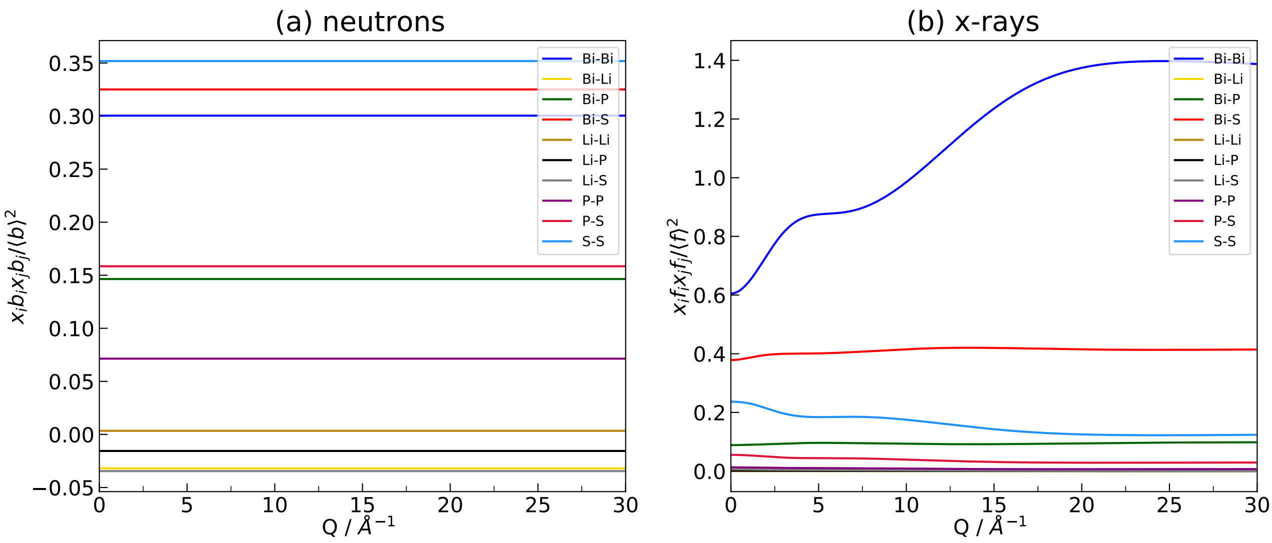

Figure S9. The respective weights of the partial pair components determined by the scattering strength and relative number of constituent elements are present for (a) neutrons and (b) X-rays. The Q-dependence of the partial weighting factors is not completely removed for X-rays due to the Morningstar-Warren approximation for multi-element compositions.

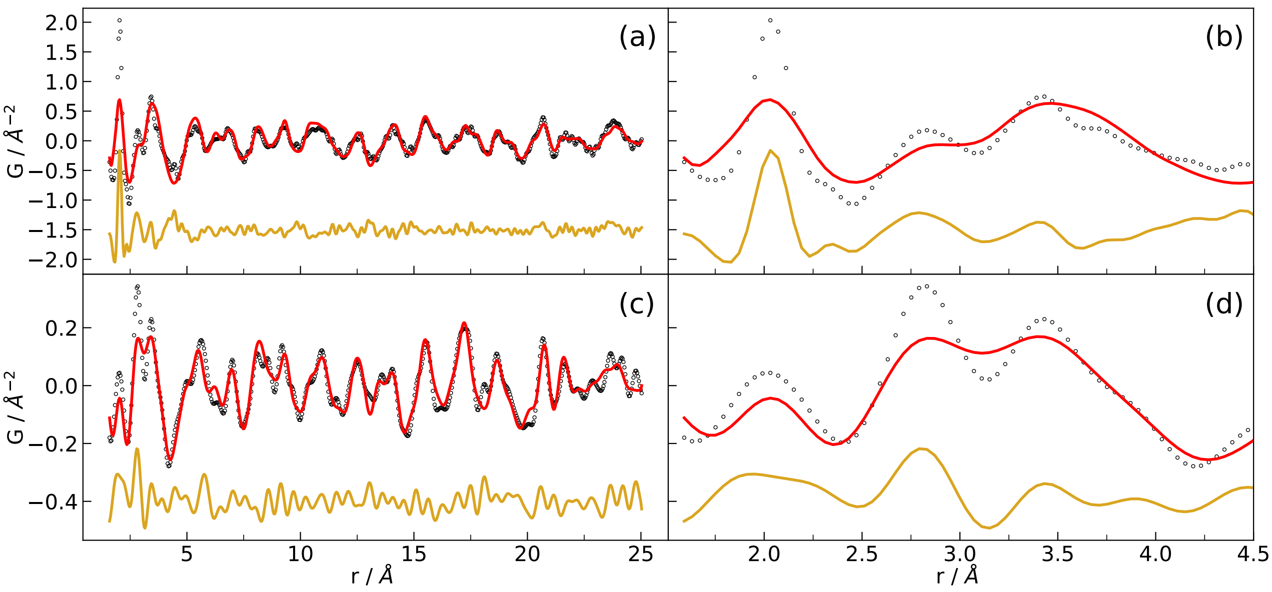

Figure S10. PDF analysis from neutron data in (a) and (b), and from X-ray data in (c) and (d) for $Li_{44.4}Bi_{21.2}(PS_4)_{36}$ compared to the simulation from the as-determined structure. The measured data (open circles), simulation from the as-determined structure (red), and difference curve (yellow). Only scale factors and a damping parameters Q_{damp} associated with instrumental broadening in reciprocal space were refined separately for each dataset.

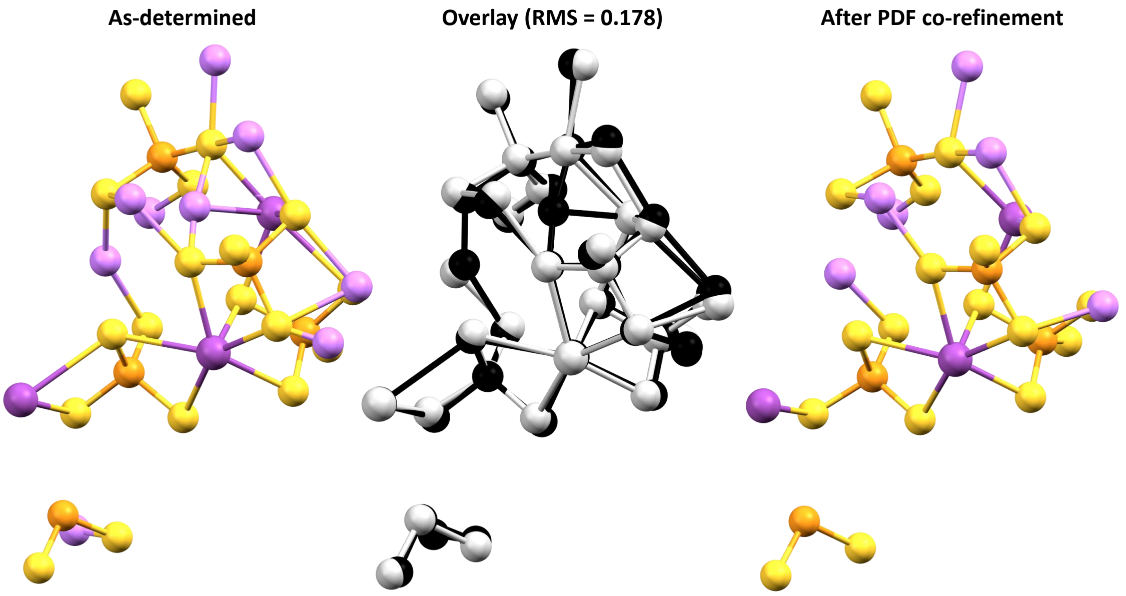

Figure S11. Comparison of the asymmetric units of the LiBiPS structure models before and after PDF refinement of the site positions. The root mean square deviation was calculated using Mercury after optimizing the overlay based upon the five phosphorus positions. The differences are primarily characterized by slight tilts and distortions of the PS_4-units. Bismuth and lithium ions are depicted in dark and bright violet, respectively, whereas phosphorus and sulfur ions are given in orange and yellow.

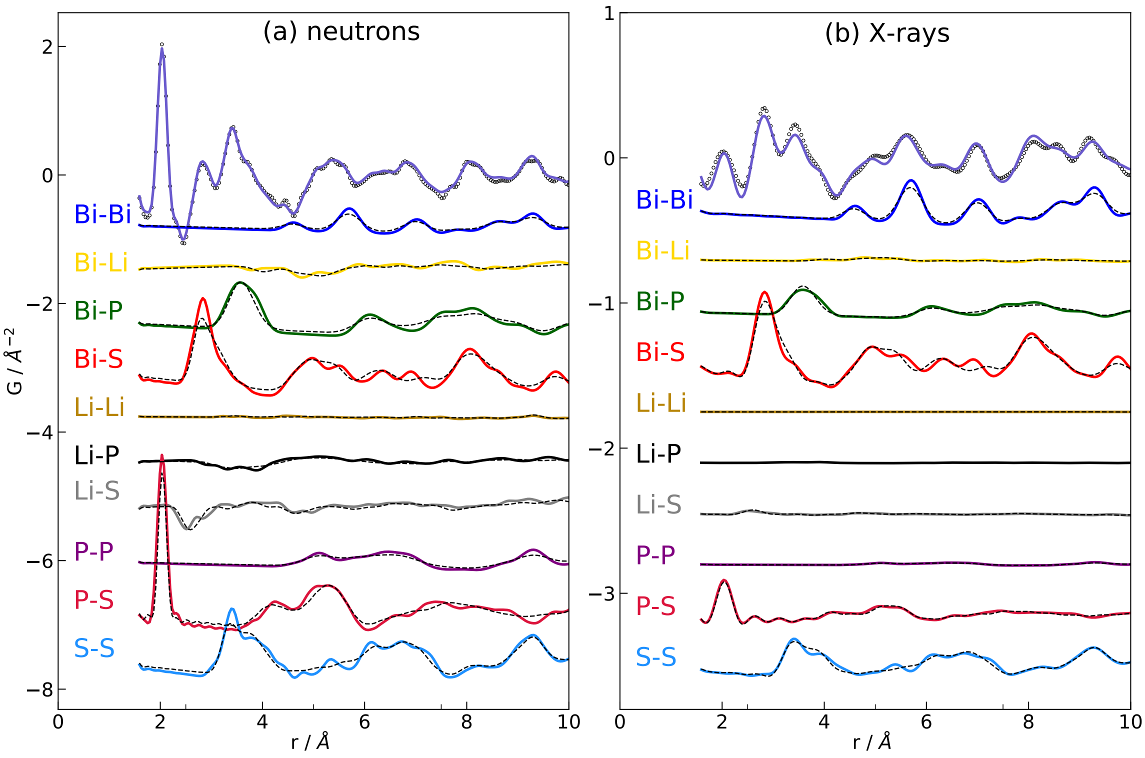

Figure S12. Partial pair contributions to the PDFs from (a) neutrons and (b) X-rays from the as-determined structure with only basic refinement (black dashed lines) and structure with site positions refined (colored lines). The dotted line at the top is the from the measurement and the colored line is the total fit after site refinement.

Table S4. Details of the models used for PDF refinement.

Parameter	No-refinement	Basic refinement	Site refinement[a]
composition	$Li_{44.4}Bi_{21.2}(PS_4)_{36}$	$Li_{44.2}Bi_{21.2}(PS_4)_{36}$	$Li_{39.28}Bi_{21.2}(PS_4)_{36}$[b]
scale (X-ray)	0.2056	0.1913	0.2058
scale (neutron)	0.4738	0.4337	0.5525
Q_{damp}[c](X-ray) [$Å^{-1}$]	0.3837	0.0233	0.0366
Q_{damp}[c](neutron) [$Å^{-1}$]	0.0492	0.0271	0.0517
a [Å]	-[d]	15.4926	15.4890
b [Å]	-	10.3071	10.3274
c [Å]	-	33.752	33.8379
β [°]	-	85.5267	85.3127
U_{iso} (Li) [$Å^2$]	-	0.01[e]	0.01[e]
U_{iso} (Bi) [$Å^2$]	-	0.0355	0.0235
U_{iso} (P) [$Å^2$]	-	0.0552	0.0313
U_{iso} (S) [$Å^2$]	-	0.0181	0.0099
δ_1 [Å]	-	1.903	1.906
R_{wp} (X-ray)	0.385	0.356	0.296
R_{wp} (neutron)	0.474	0.277	0.146

[a] See following table for values of refined site positions.
[b] Low occupancy disordered Li sites were removed to stabilize site optimization.
[c] The Q_{damp} parameters may also encode information about crystallite size, but we did not try to separate this component.
[d] Dashes indicate the value from the as-determined structure model was used.
[e] Li ADP was fixed to a value of 0.01 $Å^2$.

Table S5. Atomic positions after PDF refinement of the site positions in the as-determined model of $Li_{44.4}Bi_{21.2}(PS_4)_{36}$. The model composition is $Li_{39.28}Bi_{21.2}(PS_4)_{36}$ after removal of low occupancy disordered Li sites..

atom	Wyckoff site	x	y	z
Bi1	8f	0.69884	0.74729	0.88931
Bi2	8f	0.71508	0.67105	0.27671
Bi3	8f	0.43767	0.87737	0.95067
Li3	8f	0.43767	0.87737	0.95067
S1	8f	0.45040	0.58133	0.20431
S2	8f	0.64657	0.56757	0.94845
S3	8f	0.31389	0.79347	0.89910
S4	8f	0.73081	0.33866	0.54941
S5	8f	0.14174	0.33062	0.33923
S6	8f	0.09969	0.38566	0.00920
S7	8f	0.10912	0.62455	0.19600
S8	8f	0.45470	0.61931	0.96625
S9	8f	0.29158	0.51995	0.15945
S10	8f	0.68936	0.53866	0.14470
S11	8f	0.11571	0.60794	0.62416
S12	8f	0.65513	0.85657	0.80798
S13	8f	0.29981	0.58033	0.76493
S14	8f	0.66493	0.85025	0.48515
S15	8f	0.39933	0.20184	0.77793
S16	8f	0.00411	0.84816	0.92197
S17	8f	0.40329	0.96396	0.58673
S18	8f	0.02946	0.83780	0.37524
P1	8f	0.96892	0.03465	0.57740
P2	8f	0.32901	0.05475	0.35781
P3	8f	0.33746	0.96166	0.53577
P4	8f	0.76877	0.45805	0.18214
P5	4e	0.00000	0.19681	0.25000
Li1	8f	0.07159	0.20273	0.15378
Li2	8f	0.90015	0.13449	0.48178
Li4	8f	0.15379	0.22717	0.41989
Li5	8f	0.10099	0.85483	0.16350
Li6	8f	0.03964	0.43228	0.29750
Li7	8f	0.31451	0.40742	0.43405

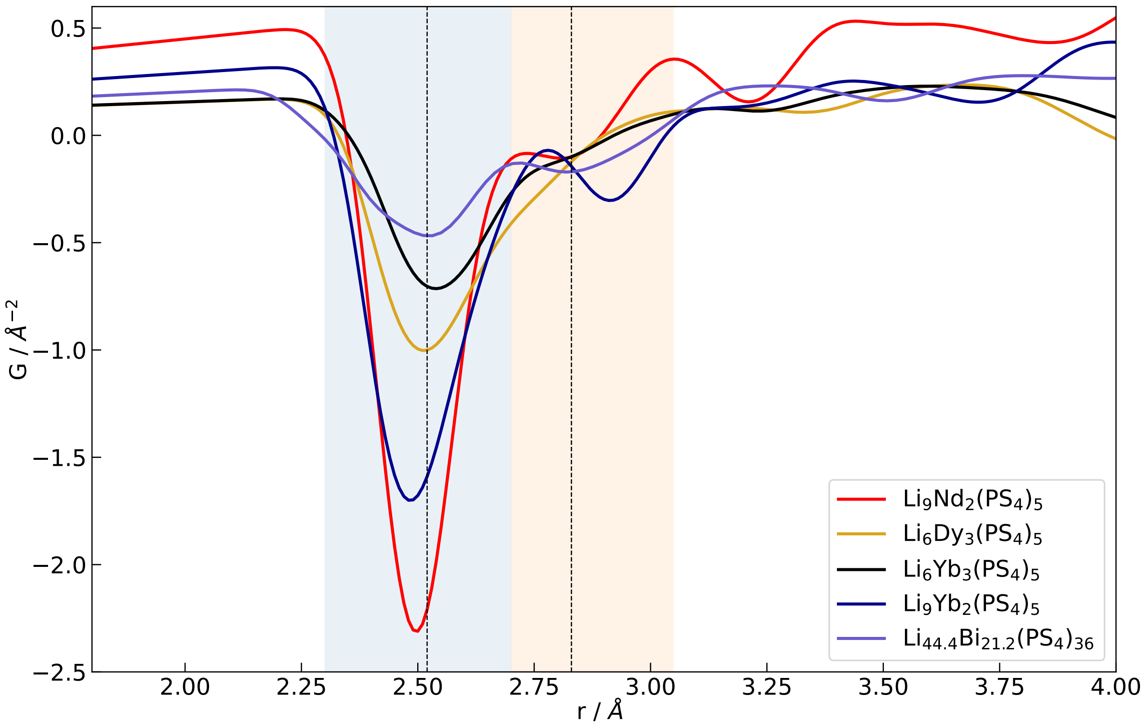

Figure S13. Comparison of the Li−S partial pair contributions for LiBiPS to that of other published structures of $Li_9Ln_2(PS_4)_5$- and $Li_6Ln_3(PS_4)_5$-type.[2–5] All published structures also show a bimodal distribution of Li−S nearest neighbor pair distances.

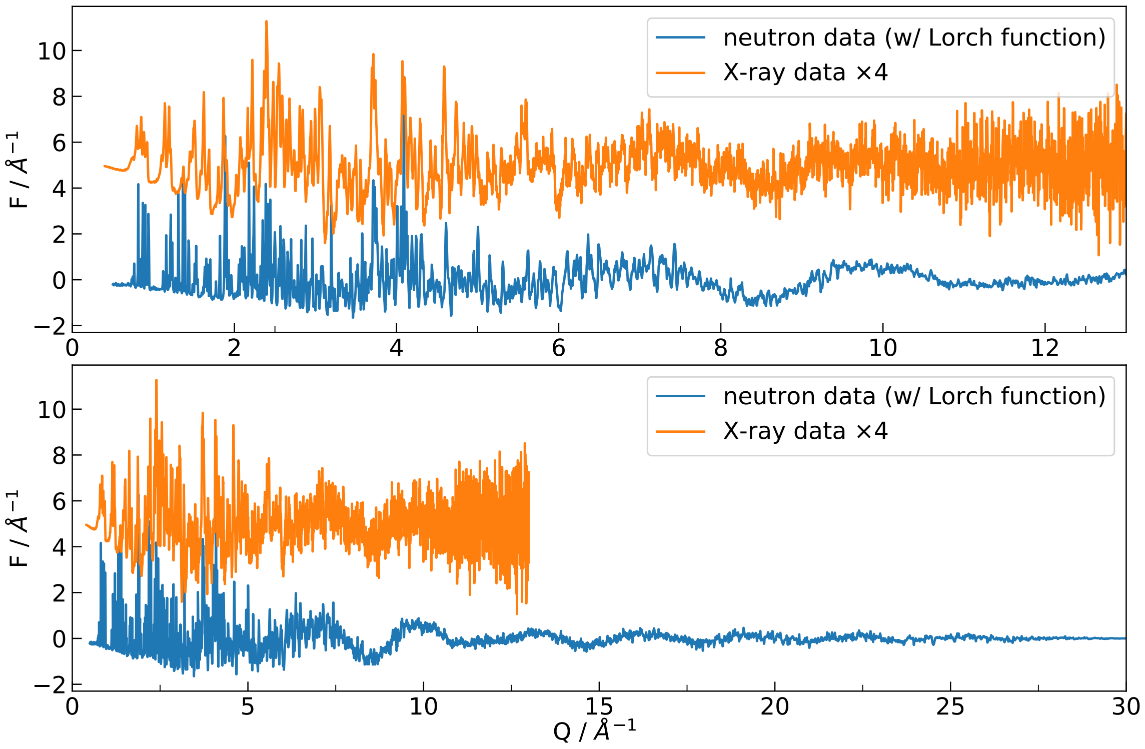

Figure S14. Comparison of reduced structure functions $F(Q)$ for the X-ray and neutron data compared over different ranges of Q.

Solid state nuclear magnetic resonance spectroscopy

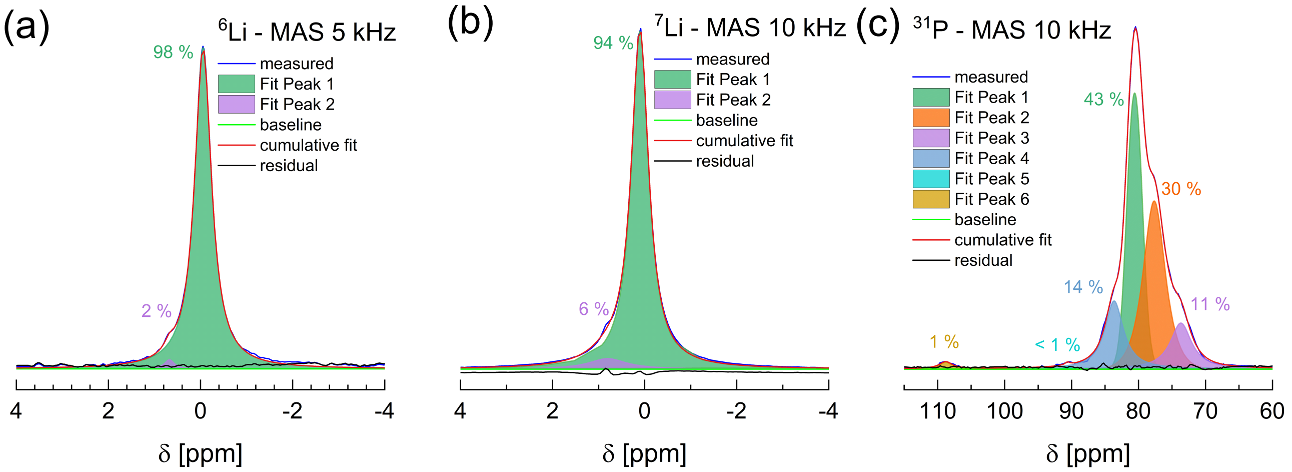

Figure S15. Experimental solid-state MAS NMR spectra of $Li_{44.4}Bi_{21.2}(PS_4)_{36}$ with deconvolution of the signals: (a) ^{6}Li-, (b) ^{7}Li- and (c) ^{31}P-ssNMR spectra.

Raman spectroscopy

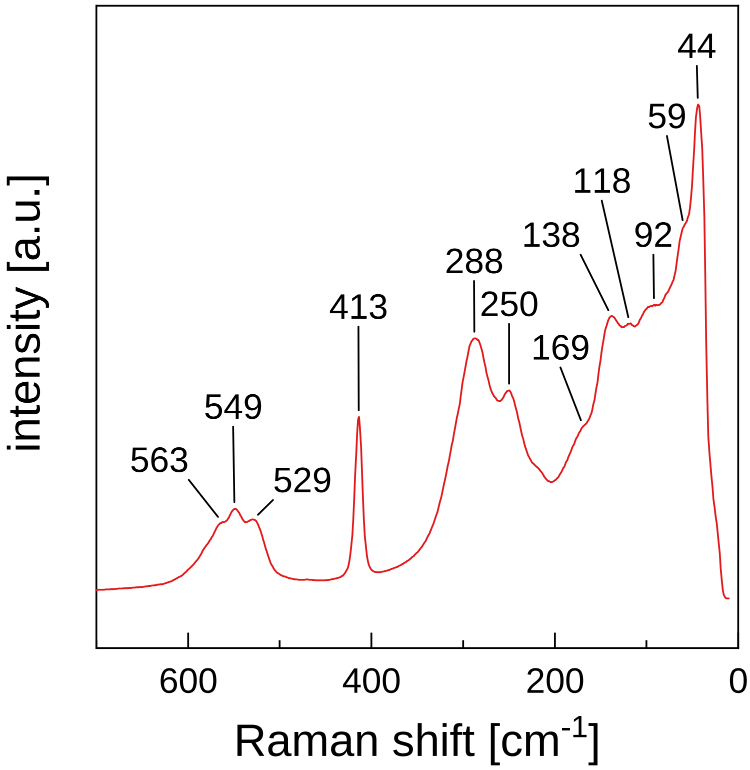

Figure S16. Raman spectrum of $Li_{45.9}Bi_{20.7}(PS_4)_{36}$, highlighting the different vibration bands. The characteristic symmetric S–P–S stretching vibration of the PS_4-tetrahedra[6] correspond to the signal at 413 cm^{-1} and the asymmetric stretching vibrations are around 549 cm^{-1}.

Electrochemical characterization

The impedance spectra at 20 °C show one wide semi-circle with a polarization tail at low frequencies. The polarization tail always was modelled with a constant phase element (CPE) in series (CPE2 and CPE3 in Figures S17a and S17b, respectively) to the other equivalent circuit elements (ECEs). The wide semi-circle can either be fitted with an equivalent circuit model (ECM) representing one big distorted semi-circle as illustrated in Figure S17a or with an ECM leading to two smaller, less distorted semi-circles as shown in Figure S17b. The semi-circles each were fitted with a resistor and a CPE in parallel. The capacitor in parallel in both ECMs was used to fit the stray capacitance originating from the setup.[7–9] Due to the smaller errors of the respective ECEs, the ECM leading to only one wide distorted semi-circle (Figure S17a) was used for fitting the data above −10 °C. To see whether

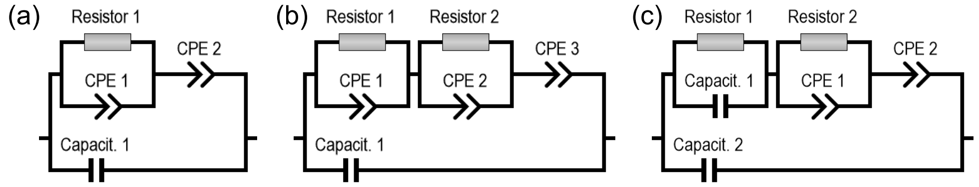

Figure S17. Different equivalent circuit models used for fitting the impedance data: (a) ECM I, (b) ECM II and (c) ECM III.

this wide distorted semi-circle is actually a convolution of two distinct processes, low temperature electrochemical impedance spectroscopy (EIS) at −76 °C was conducted, where the two semi-circles clearly can be differentiated from one another, and thus the ECM given in Figure S17b can be used for fitting the impedance data at low temperatures. According to the Rietveld refinement of the powder X-ray diffraction (PXRD) data at 100 K shown in Figure S3, any phase transition during the EIS measurements at low temperatures can be excluded. The values of the respective ECEs of the different ECMs are given in Table S6. According to the Brug capacitances of ECM II (Figure S17b) at −76 °C the first semi-circle (4.4×10^{-12} F) at higher frequencies can be ascribed to a bulk and the second semi-circle (5.2×10^{-11} F) at lower frequencies to a grain boundary related process.[10] Similar observations have been made for the platinum sputtered samples, where contact related contributions are less severe. An exemplary fit of the impedance spectra at −10 °C is given in Figure S21 and the values of the different ECEs are summarized in Table S7. Here, similar to ECM II two different processes can be differentiated, but the grain boundary related process (semicircle at lower frequencies having a capacitance of 1.8×10^{-10} F) could only be resolved with a resistor and a capacitor in parallel (see ECM III in Figure S17c) at this temperature.

While the electronic conductivity can be obtained from the measured equilibrium current I_{eq} at a certain potential U, the ionic conductivity in direct current (DC) polarization measurements is derived from the measured initial current I_{init} and the specific resistivity ρ, which depends on the dimensions (thickness d and area A) of the measured pellet.[11,12]

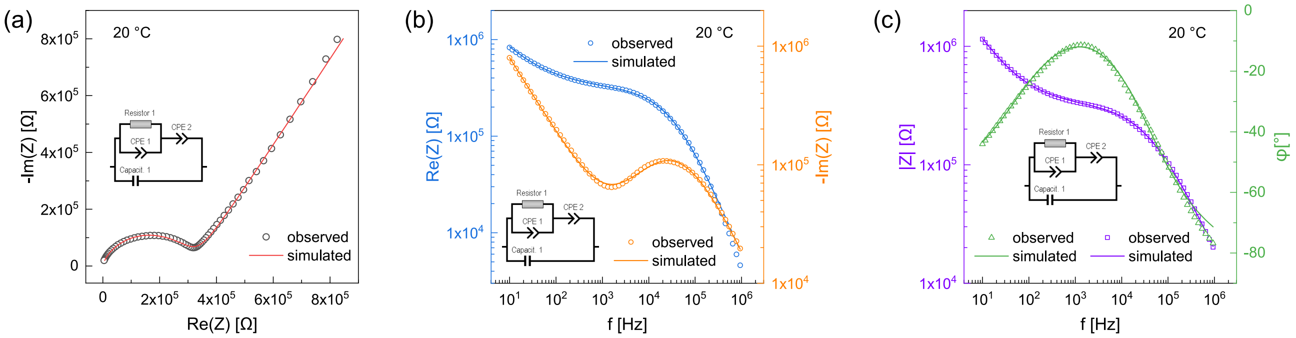

Figure S18. Nyquist and Bode plots of $Li_{45}Bi_{21}(PS_4)_{36}$ at 20 °C in (a) to (c) using ECM I.

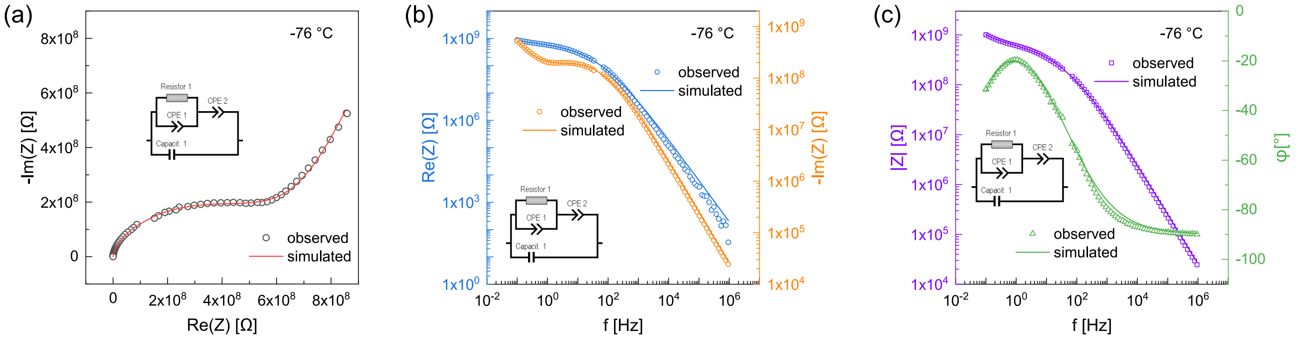

Figure S19. Nyquist and Bode plots of $Li_{45}Bi_{21}(PS_4)_{36}$ at −76 °C in (a) to (c) using ECM I.

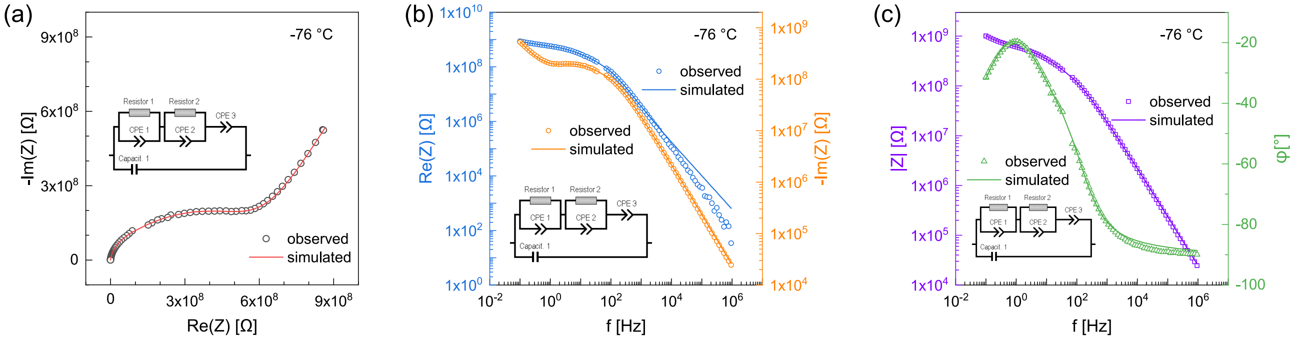

Figure S20. Nyquist and Bode plots of $Li_{45}Bi_{21}(PS_4)_{36}$ at −76 °C in (a) to (c) using ECM II.

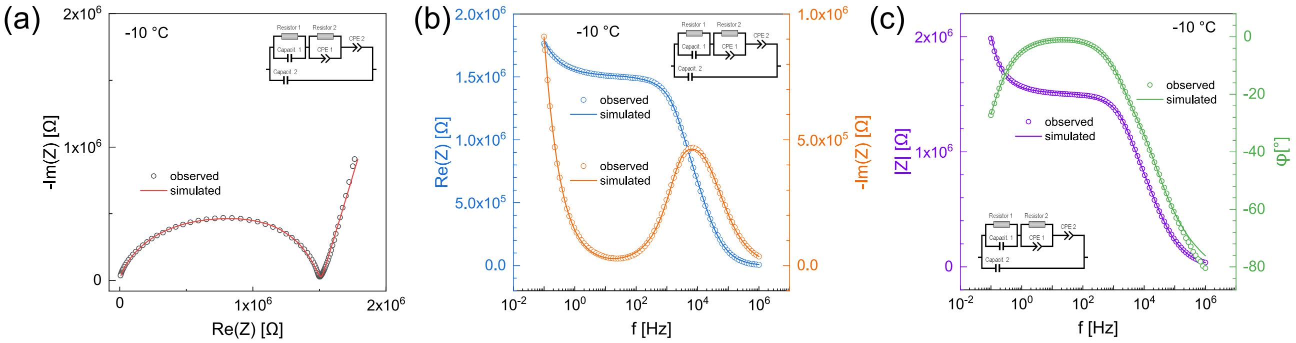

Figure S21. Nyquist and Bode plots of $Li_{45}Bi_{21}(PS_4)_{36}$ at −10 °C and sputtered with platinum in (a) to (c) using ECM III.

Table S6. Values of the different equivalent circuit elements obtained from the fittings depicted in Figures S18–S20 for the equivalent circuit models I & II (shown in Figures S17a and S17b) for $Li_{45}Bi_{21}(PS_4)_{36}$. Note that Q_2 and α_2 of ECM I corresponds to the polarization tail, whereas this is the case for Q_3 and α_3 in ECM II.

ECEs	unit	I at 20 °C	I at −76 °C	II at −76 °C
R_1	[Ω]	3.24×10^5	7.36×10^8	1.38×10^8
Q_1	[C]	1.15×10^{-9}	2.21×10^{-10}	2.60×10^{-11}
α_1		0.64	0.48	0.76
$C_{Brug}^{Q_1}$	[F]	1.30×10^{-11}	3.08×10^{-11}	4.43×10^{-12}
R_2	[Ω]	–	–	4.54×10^8
Q_2	[C]	7.69×10^{-8}	2.77×10^{-9}	1.81×10^{-10}
α_2		0.63	0.76	0.67
$C_{Brug}^{Q_2}$	[F]	–	–	5.23×10^{-11}
Q_3	[C]	–	–	2.32×10^{-9}
α_3		–	–	0.67
C_{stray}	[F]	4.52×10^{-12}	6.20×10^{-12}	6.13×10^{-12}

Table S7. Values of the different equivalent circuit elements obtained from the fittings depicted in Figure S21 for the equivalent circuit model III (shown in Figure S17c) for platinum sputtered $Li_{45}Bi_{21}(PS_4)_{36}$.

ECEs	unit	III at −10 °C
R_1	[Ω]	2.99×10^5
C_1	[F]	1.84×10^{-10}
R_2	[Ω]	1.22×10^6
Q_1	[C]	4.93×10^{-10}
α_2		0.64
$C_{Brug}^{Q_1}$	[F]	7.20×10^{-12}
Q_2	[C]	1.54×10^{-6}
α_2		0.81
C_{stray}	[F]	2.74×10^{-12}

$$\sigma = \rho^{-1} = \frac{d}{AR} \qquad \text{(S5)}$$

$$I_{eq} = U/R_{el} \qquad \text{(S6)}$$

$$I_{init} = U/R_{tot} \qquad \text{(S7)}$$

$$\frac{1}{R_{tot}} = \frac{1}{R_{el}} + \frac{1}{R_{ion}} \qquad \text{(S8)}$$

The ionic transference number t_{ion} can be calculated from the ionic and electronic conductivity and is close to unity for good ionic conductors.

$$t_{ion} = \frac{\sigma_{ion}}{\sigma_{ion} + \sigma_{el}} \qquad \text{(S9)}$$

Furthermore, the conductivity diffusion coefficient D_{σ}^{DC} can be estimated from Equation S10, where z and n refer to the charge and number of the mobile charge carriers per unit cell volume, e to the elementary charge, k_B to the Boltzmann constant, T to the temperature and μ_{ion} and σ_{ion} to the mobility and to the conductivity of the ionic species, respectively.[13,14]

$$D_{\sigma}^{\mathrm{DC}} = \frac{k_B T \mu_{ion}^{\mathrm{DC}}}{ze} = \frac{k_B T \sigma_{ion}^{\mathrm{DC}}}{nz^2e^2} \tag{S10}$$

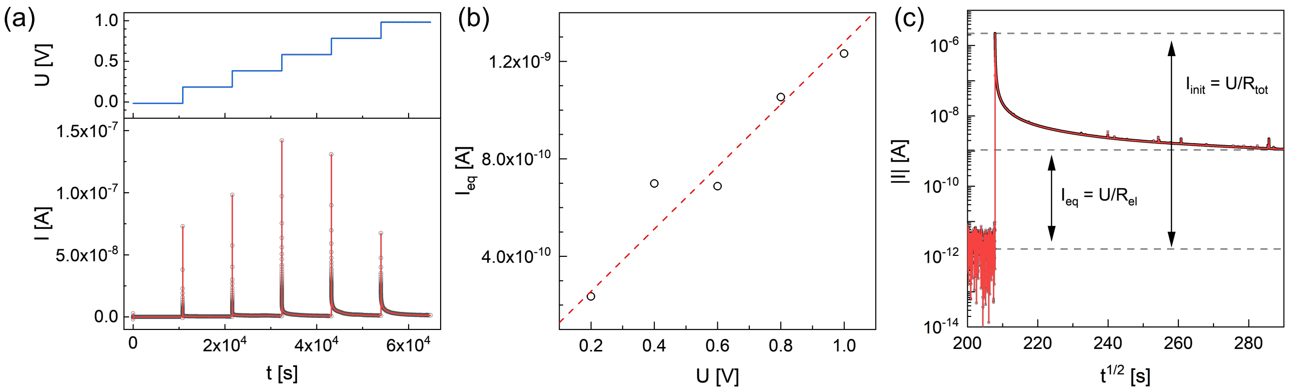

Figure S22. Results of the direct current (DC) polarization measurements for $Li_{44.4}Bi_{21.2}(PS_4)_{36}$: (a) DC polarization, (b) the resulting current against voltage plot and (c) fast DC measurement.

Pulsed field gradient NMR

The tracer diffusion coefficient D_{tr}^{NMR} obtained from pulsed field gradient nuclear magnetic resonance (PFG-NMR) is connected to the uncorrelated diffusion coefficient D_{uc}^{NMR} according to Equation S11, where the Bardeen-Herring tracer correlation factor is given with $f \leq 1$.[15,16]

$$D_{tr}^{\mathrm{NMR}} = f D_{uc}^{\mathrm{NMR}} \tag{S11}$$

The mean diffusion length is approximated *via* the mean squared displacement $\sqrt{\langle x^2 \rangle}$ and can be estimated from the used diffusion time Δ^{NMR} and $f = 1$ according to Einstein-Smoluchowski relation given in Equation S12.[17,18]

$$\sqrt{\langle x^2 \rangle} = \sqrt{2 D_{uc}^{\mathrm{NMR}} \Delta^{\mathrm{NMR}}} \tag{S12}$$

The diffusion jump rate τ^{-1} can be calculated according to Equation S13 using the length of a jump $l = 2.219$ Å obtained from *softBV* calculations.[16]

$$\tau^{-1} = \frac{6 D_{tr}^{\mathrm{NMR}}}{l^2 f} \tag{S13}$$

Furthermore, the ionic conductivity can be estimated using the Nernst-Einstein approximation given in Equation S14, where H_R corresponds to the Haven ratio,[16] which is given in Equation S15 and connects the tracer diffusion coefficient and the conductivity diffusion coefficient D_{σ}^{DC} with one another.

$$\sigma_{ion}^{\mathrm{NMR}} = \frac{D_{tr}^{\mathrm{NMR}} n z^2 e^2}{k_B T H_R} \tag{S14}$$

$$H_R = \frac{D_{tr}^{\mathrm{NMR}}}{D_{\sigma}^{\mathrm{DC}}} \tag{S15}$$

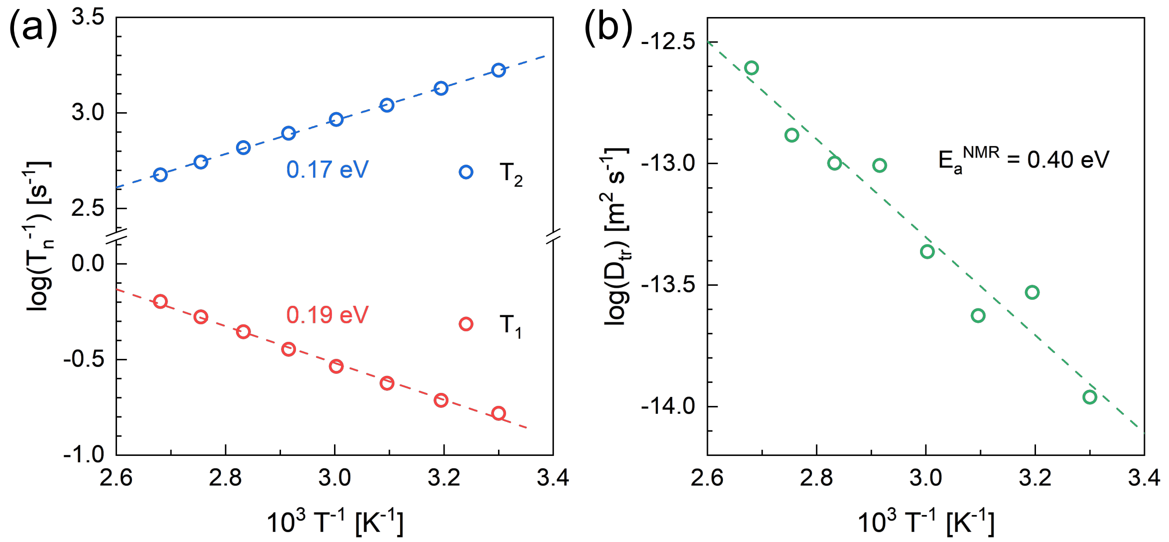

Figure S23. Results of the temperature dependent ^{7}Li spin-lattice (T_1) and spin-spin (T_2) relaxation measurements in (a) and the resulting tracer diffusion coefficient in (b) of $Li_{44.4}Bi_{21.2}(PS_4)_{36}$.

Bond valence sum calculations

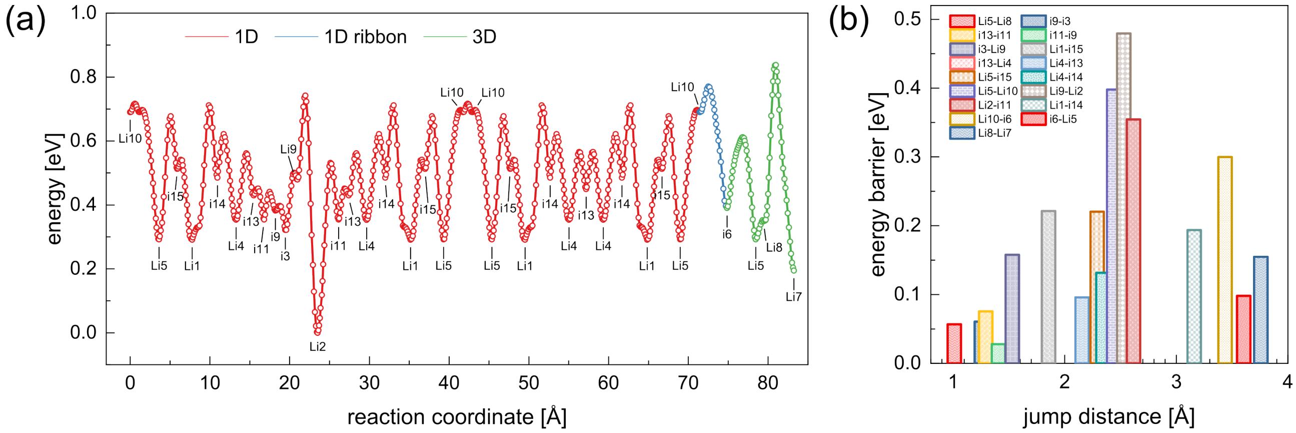

Figure S24. Results of the BVS calculations: (a) Energies of different lithium and interstitial sites as well as saddle points. The red colour denotes 1D-, blue 1D zig-zag-/ribbon-like and green 3D-trajectories. In (b) the energy barriers (corresponds to the energy difference between two sites) and the length of the different site-to-site jumps are given.

References

(1) Toby, B. H. *Powder Diffraction* **2006**, *21*, 67–70.

(2) Komm, T.; Schleid, T. *J. Alloys Compd.* **2006**, *418*, 106–110.

(3) Müller, C.; Jörgens, S.; Mewis, A. *Z. Anorg. Allg. Chem.* **2007**, *633*, 1633–1638.

(4) Lange, P. L.; Schleid, T. *Z. Naturforsch.* **2021**, *76*, 281–291.

(5) Lange, P. L.; Komm, T.; Schleid, T. *Z. Anorg. Allg. Chem.* **2021**, *647*, 2113–2121.

(6) Tiwari, D.; Alibhai, D.; Cherns, D.; Fermin, D. J. *Chem. Mater.* **2020**, *32*, 1235–1242.

(7) Kuhn, A.; Duppel, V.; Lotsch, B. V. *Energy Environ. Sci.* **2013**, *6*, 3548–3552.

(8) Scholz, T.; Schneider, C.; Eger, R.; Duppel, V.; Moudrakovski, I.; Schulz, A.; Nuss, J.; Lotsch, B. V. *J. Mater. Chem. A* **2021**, *9*, 8692–8703.

(9) Balabajew, M.; Roling, B. *Electrochim. Acta* **2015**, *176*, 907–918.

(10) Irvine, J. T. S.; Sinclair, D. C.; West, A. R. *Adv. Mater.* **1990**, *2*, 132–138.

(11) Joachim Maier, *Physical Chemistry of Ionic Materials: Ions and Electrons in Solids*; John Wiley & Sons, Ltd: Chichester, 2004.

(12) Senocrate, A.; Moudrakovski, I.; Kim, G. Y.; Yang, T.-Y.; Gregori, G.; Grätzel, M.; Maier, J. *Angew. Chemie* **2017**, *129*, 7863–7867.

(13) Einstein, A. *Annalen der Physik* **1905**, *322*, 549–560.

(14) von Smoluchowski, M. *Annalen der Physik* **1906**, *326*, 756–780.

(15) Bardeen, J.; Herring, C. In *Imperfections in Nearly Perfect Crystals*; Shockley, W., Hollomon, J. H., Mauerer, R., Seitz, F., Eds.; John Wiley & Sons, Inc., 1952; Chapter Diffusion in Alloys and the Kirkendall Effect, pp 261–288.

(16) Murch, G. E. *Solid State Ionics* **1982**, *7*, 177–198.

(17) Islam, M. A. *Phys. Scr.* **2004**, *70*, 120–125.

(18) Kuhn, A.; Gerbig, O.; Zhu, C.; Falkenberg, F.; Maier, J.; Lotsch, B. V. *Phys. Chem. Chem. Phys.* **2014**, *16*, 14669–14674.
